# Pharmacological inhibition of IL12β is effective in treating pressure overload-induced cardiac inflammation and heart failure

**DOI:** 10.3389/fimmu.2025.1624940

**Published:** 2025-08-15

**Authors:** Umesh Bhattarai, Xiaochen He, Ziru Niu, Lihong Pan, Dongzhi Wang, Hao Wang, Heng Zeng, Jian-Xiong Chen, Joshua S. Speed, John S. Clemmer, Yingjie Chen

**Affiliations:** ^1^ Department of Physiology and Biophysics, School of Medicine, University of Mississippi Medical Center, Jackson, MS, United States; ^2^ Department of Pharmacology and Toxicology, School of Medicine, University of Mississippi Medical Center, Jackson, MS, United States

**Keywords:** IL12β, inflammation, heart failure, T cells, macrophages, lung remodeling

## Abstract

**Background and objective:**

Emerging evidence indicates that inflammation regulates cardiac remodeling and heart failure (HF). IL12β is a subunit for proinflammatory cytokines IL12 and IL23. However, the effect of IL12β inhibition on HF development and the underlying mechanism is not understood.

**Methods:**

We determined the effect of pharmacological inhibition of IL12β using IL12β blocking antibody on transverse aortic constriction (TAC)-induced left ventricular (LV) inflammation and HF development.

**Results:**

IL12β blocking antibody significantly attenuated TAC-induced LV immune cell infiltration, hypertrophy, fibrosis, dysfunction, and the consequent pulmonary inflammation and remodeling. More specifically, we found that IL12β blocking antibody significantly attenuated TAC-induced LV and pulmonary infiltration of neutrophils, macrophages, CD11c^+^ dendritic cells, CD8^+^ T cells, and CD4^+^ T cells. Moreover, IL12β blocking antibody significantly suppressed the production of pro-inflammatory cytokine pro-IL1β and IFNγ by macrophages and IFNγ by CD8^+^ T cells and/or CD4^+^ T cells.

**Conclusions:**

These findings indicate that pharmacological inhibition of IL12β effectively protected the heart from systolic overload-induced inflammation, remodeling, and dysfunction by reducing the proinflammatory signaling from both innate and adaptive immune responses.

## 1 Introduction

Heart Failure (HF) or left ventricular (LV) failure is a pathological condition in which the heart is unable to pump enough nutrients and oxygen-rich blood throughout the systemic circulation to meet the body’s needs. Although significant progress has been made in its diagnosis and treatment, HF remains one of the major public health problems and leads to significant cardiovascular morbidity and mortality worldwide (1). Chronic pressure overload conditions, such as hypertension, are one of the major risk factors for HF. An increase in afterload and cardiac pressure causes LV hypertrophy, LV failure, and the consequent WHO class 2 pulmonary hypertension and right ventricular (RV) hypertrophy. The transitional process from LV failure to HF-induced lung remodeling and RV failure is often termed as HF progression (2–6). Despite significant progress in HF diagnosis and treatment, current therapies remain suboptimal in preventing disease progression. Thus, identifying potential new therapeutic targets for HF treatment is highly significant.

Both clinical and experimental evidence suggest that inflammation promotes the development and progression of HF, while inhibition of inflammation can effectively suppress the development and progression of hypertension and HF (7–13). For example, previous studies showed that pro-inflammatory cytokines such as tumor necrosis factor α (TNFα) and interleukin 1β (IL1β) are increased in heart and blood samples of HF patients (14, 15) and experimental HF models (16, 17). A clinical trial targeting IL1β using canakinumab at a dose of 150 mg every 3 months led to a significantly lower rate of recurrent cardiovascular events than placebo in patients with previous myocardial infarction (18). In addition, inhibition of IL1β significantly reduced transverse aortic constriction (TAC)-induced LV hypertrophy, dysfunction, and HF progression (10). Furthermore, studies from our lab and others demonstrated that cardiac inflammation and HF development are promoted by immune cell subsets including CD4^+^ T cells (19), CD8^+^ T cells (20), and NK1.1^+^ lymphatic cells (9), at least partially through modulating IFNγ signaling in mice. Meanwhile, CD11c^+^ dendritic cells (DCs) (21) and macrophages (22) also promoted TAC-induced cardiac inflammation and HF development partially through modulating T cell activation and IL1β signaling in mice. Moreover, we found that TAC-induced HF in mice is associated with T cell activation, while TAC-induced LV hypertrophy and HF are significantly reduced by inhibition of T cell activation through CD28 knockout, CD80/CD86 double knockout, or administration of Ctla4 Ig in mice (11). Our previous studies also demonstrated that chronic HF causes significant lung inflammation as indicated by increased immune cell infiltration and activation, and increased production of pro-inflammatory cytokines by the infiltrated immune cells (2, 23, 24). However, as most of these investigated targets are not currently used clinically, identifying therapeutic targets that can be potentially regulated in the clinical setting may lead to novel HF therapies.

IL12β is a subunit for IL12 and IL23, two important proinflammatory cytokines mainly produced by activated macrophages and DCs (25–29). Inhibition of IL12β can simultaneously suppress the proinflammatory effects of IL12 and IL23 and their downstream targets. For example, IL12 promotes IFNγ production by T cells and NK cells, and inhibition of IL12β can attenuate cardiac IL12 and IFNγ signaling. IL12 plays an important role in promoting the proliferation, activation, and mobilization of CD8^+^ T cells, CD4^+^ Th1 cells, and NK cells (25, 30–40), a group of immune cells that promote cardiac inflammation and HF in experimental animals secondary to systolic overload produced by TAC (9, 19, 20). Previous studies from us and others found that TAC-induced HF is associated with increased cardiopulmonary IFNγ^+^CD4 T cells, IFNγ^+^CD8 T cells, and IFNγ^+^NK cells (9, 41). Meanwhile, IL23 plays an important role in stimulating IL17 production by Th17 cells or γδT17 cells through activating IL23 receptors expressed on these immune cells through *a* retinoic acid orphan receptor gamma (RORγ)-dependent pathway, a signaling pathway where RORγ and RORγt (a nuclear receptor protein) influence gene expression, cell proliferation and function mainly in Th17 cells and γδT17 cells of the immune system (27, 28, 42–46). Inhibition of IL12β also attenuates the IL23 and IL17 signaling pathway, another pathway that promotes cardiac inflammation and HF (47). Thus, inhibition of IL12β can effectively attenuate both IL12/IFNγ and IL23/IL17 axes. Importantly, the IL12β blocking antibody (anti-IL12β antibody), ustekinumab, is currently used clinically to treat inflammatory bowel disease, psoriasis, and psoriatic arthritis. We propose that inhibiting IL12β signaling may be potentially beneficial to patients with HF. Therefore, the central hypothesis of this study is that pharmacological blockade of IL12β signaling will attenuate cardiac inflammation and HF development during chronic pressure overload. Here, we investigated whether treatment with IL12β blocking antibody attenuates TAC-induced LV inflammation, fibrosis, and HF in mice.

## 2 Materials and methods

### 2.1 Mice and experimental protocols

Wild-type C57BL/6J (Jackson Lab, Strain #000664) female mice were used for sham or TAC surgery, a commonly used surgical procedure to mimic clinical conditions such as hypertension or aortic stenosis. Pharmacological inhibition of IL12β was achieved by anti-IL12β antibody (BioXCell, BE0051). Briefly, one week after the initial cardiac functional test by echocardiography, C57BL/6J mice were subjected to either sham surgery or TAC. The TAC surgery was performed using a 27-gauge blunt needle after anesthetizing the mice with an intraperitoneal injection of ketamine (100 mg/Kg) and xylazine (10 mg/Kg) as previously described (48, 49). The control mice used in this study are sham-operated mice without anti-IL12β treatment. After TAC surgery, mice were randomly divided into two groups and intraperitoneally injected with 250 µg/mouse of either anti-IL12β antibody or control IgG every 3 days for 4 weeks. The dose of anti-IL12β antibody is according to previous reports with minor modification (50, 51). Bodyweight gain/loss was monitored weekly, and LV function was monitored every two weeks. Samples were collected at 4 weeks after TAC. Pulmonary function was determined before the sample collection. Cardiac and pulmonary tissues were harvested and utilized for subsequent histological, immuno-histological, and biochemical analyses. All mice were housed in a temperature-controlled environment with 12-hour light/dark cycles. This study was approved by the Institutional Animal Care and Use Committee at the University of Mississippi Medical Center.

### 2.2 Echocardiography

Echocardiography was performed using a VisualSonics Vevo 3100 imaging system (FUJIFILM VisualSonics Inc., Canada) as previously described (49). Briefly, the mice were anesthetized by inhalation of 1-2% isoflurane mixed with 100% oxygen. M-mode echocardiographic images were taken and analyzed using Vevo LAB software (FUJIFILM VisualSonics Inc., Canada) to measure heart rate, LV ejection fraction, LV fractional shortening, LV end-systolic diameter, LV end-systolic volume, LV end-diastolic diameter, LV end-diastolic volume, LV anterior and posterior wall thickness at end-systole or end-diastole, stroke volume, and cardiac output.

### 2.3 Histological staining

Histological and immunological staining were performed according to previous studies (52, 53). Briefly, cardiac and pulmonary samples were fixed in 4% formaldehyde, embedded in paraffin blocks, and sections of 5 µm were sliced and placed on glass slides. The tissue sections were deparaffinized and rehydrated. Sirius Red/Fast Green Staining Kit (Chondrex Inc., 90461) was used for LV and lung fibrosis staining. Alexa Flour-488 conjugated wheat germ agglutinin (WGA) (Invitrogen, W11261, 5 µg/mL) staining was used to measure LV cardiomyocyte cross-sectional area. The cross-sectional area of 100 LV cardiomyocytes was measured and averaged to get the mean cardiomyocyte cross-sectional area. Relative expression of β-myosin heavy chain (β-MHC) in LV and RV tissues was determined by using mouse anti-β-MHC antibody (R&D Systems, MAB4470, 5µg/mL) and Alexa Flour-555 conjugated goat anti-mouse secondary antibody (Invitrogen, A21424, 1:1000 dilution). Fibrosis, LV cardiomyocyte cross-sectional area, and relative β-MHC expression were quantified using ImageJ software from the National Institutes of Health. The infiltrated CD45^+^ leukocytes in the LV and lung tissues were stained for visualization with goat anti-CD45 antibody (R&D Systems, AF114, 1:100 dilution) and Alexa Flour-555 conjugated donkey anti-goat secondary antibody (Invitrogen, A21432, 1:1000 dilution). Muscularization of pulmonary arterioles was determined by using mouse anti-α smooth muscle actin (αSMA) (Invitrogen, 14-9760-82, 1:200 dilution) and rabbit anti-CD31 antibody (Cell Signaling Technologies Inc., 77699, 1:200 dilution) and Alexa Flour-555 conjugated goat anti-mouse (Invitrogen, A21424, 1:1000 dilution) and Alexa Flour-594 conjugated goat anti-rabbit (Invitrogen, A11012, 1:1000 dilution) secondary antibodies, respectively for visualization. The tissue sections were then mounted with 4’,6-diamidino-2-phenylindole (DAPI) containing mounting media (enQuire BioReagents, QS4-20ML) to visualize the nucleus. All the histological images were captured using Mantra Quantitative Pathology Imaging System (Perkin Elmer) and infiltrated CD45^+^ leukocytes were quantified using inForm software version 2.2.1 (Perkin Elmer).

### 2.4 Lung function measurement

Mice were anesthetized with an intraperitoneal injection of ketamine (100 mg/Kg) and xylazine (10 mg/Kg). A tracheostomy was then performed, and an 18G metal cannula was inserted into the trachea and secured in place with a suture around the trachea. The mice were then connected to a small animal ventilator (flexiVent, SCIREQ, Canada) and subjected to mechanical ventilation at a rate of 150 breaths per minute, with a tidal volume of 10 mL/Kg and positive end-expiratory pressure of 3 cmH_2_O (54). The forced oscillation technique (FOT) was utilized, incorporating both the single-frequency FOT (“Snapshot-150 perturbation”) and the multi-frequency FOT (“Quick Prime-3 perturbation”). Data from single-frequency FOT were analyzed using a single-compartment model to determine respiratory system resistance and compliance. Data from multi-frequency FOT were analyzed with a constant phase to determine tissue damping and tissue elastance. The pressure-volume loops were generated to measure quasi-static compliance and inspiratory capacity.

### 2.5 Flow cytometry analyses

Lung tissue was harvested after perfusing the tissue with cold PBS through the right ventricle and digested in Hank’s balanced salt solution (HBSS) (Life Technologies Corporation) supplied with 1mg/ml collagenase D (Roche Diagnostics, Germany) at 37°C for 30 minutes using a tissue dissociator (Miltenyi Biotec) as previously described (23). The cell suspension was then passed through a 100 µm cell strainer to remove debris. The red blood cells in the filtered cell suspension were then lysed with 2 mL RBC lysis buffer (Life Technologies Corporation). The cells were then stained with fixable viability dye (BD Bioscience, FVS440UV) in PBS at 4°C for 30 minutes. After washing the cells with 2 mL staining buffer, the cells were then incubated with CD16/32 antibody (Biolegend, clone 2.4G2) at 4°C for 30 minutes to block the non-specific binding of antibodies to FcRγ. The cells were then stained with fluorescent conjugated multi-staining antibodies (**Supplementary Table 1**) at 4°C for 30 minutes. For cytokine production assay, the isolated immune cells were stimulated with 1X Cell Stimulation Cocktail (Invitrogen, 00-4970-93) and 1X Protein Transport Inhibitor Cocktail (Invitrogen, 00-4980-93) in RPMI 1640 with 10% FBS at 37°C for 2 hours. The cells were then stained with antibodies for cell surface markers, permeabilized, and stained with antibodies against different intracellular cytokines (**Supplementary Table 1**). Data were acquired on a BD FACSymphony™ A3 Cell Analyzer (BD Biosciences) and analyzed by using FlowJo-v10 (FlowJo, OR) software. The gating strategies used for lung flow cytometry analysis are presented in **Supplementary Figure 1**. For the flow cytometry of LV tissue, LV tissues were minced into small pieces and digested in HBSS supplied with Deoxyribonuclease I (66.7 μg/mL, Sigma-Aldrich) and LiberaseTM (125 μg/mL, Roche Diagnostics, Germany) at 37°C for 30 minutes using a tissue dissociator (Miltenyi Biotec). The staining procedure is the same as described above. The gating strategies used for heart flow cytometry analysis are presented in **Supplementary Figure 2**. The total number of different immune cells per LV was determined by knowing the amount of LV tissue used for flow cytometry, total LV weight, total number of cells present in the digested tissue suspension, and percentage of each immune cell subset in the total stained cells.

### 2.6 Statistical analysis

Data are presented as Mean ± SEM. One-way ANOVA followed by Bonferroni *post-hoc* test was utilized to test statistical differences between 3 groups. All the statistical tests were performed using GraphPad Prism 10 software. p<0.05 was considered statistically significant.

## 3 Results

### 3.1 Pharmacological inhibition of IL12β attenuated TAC-induced LV dysfunction in mice

**Figure 1:**
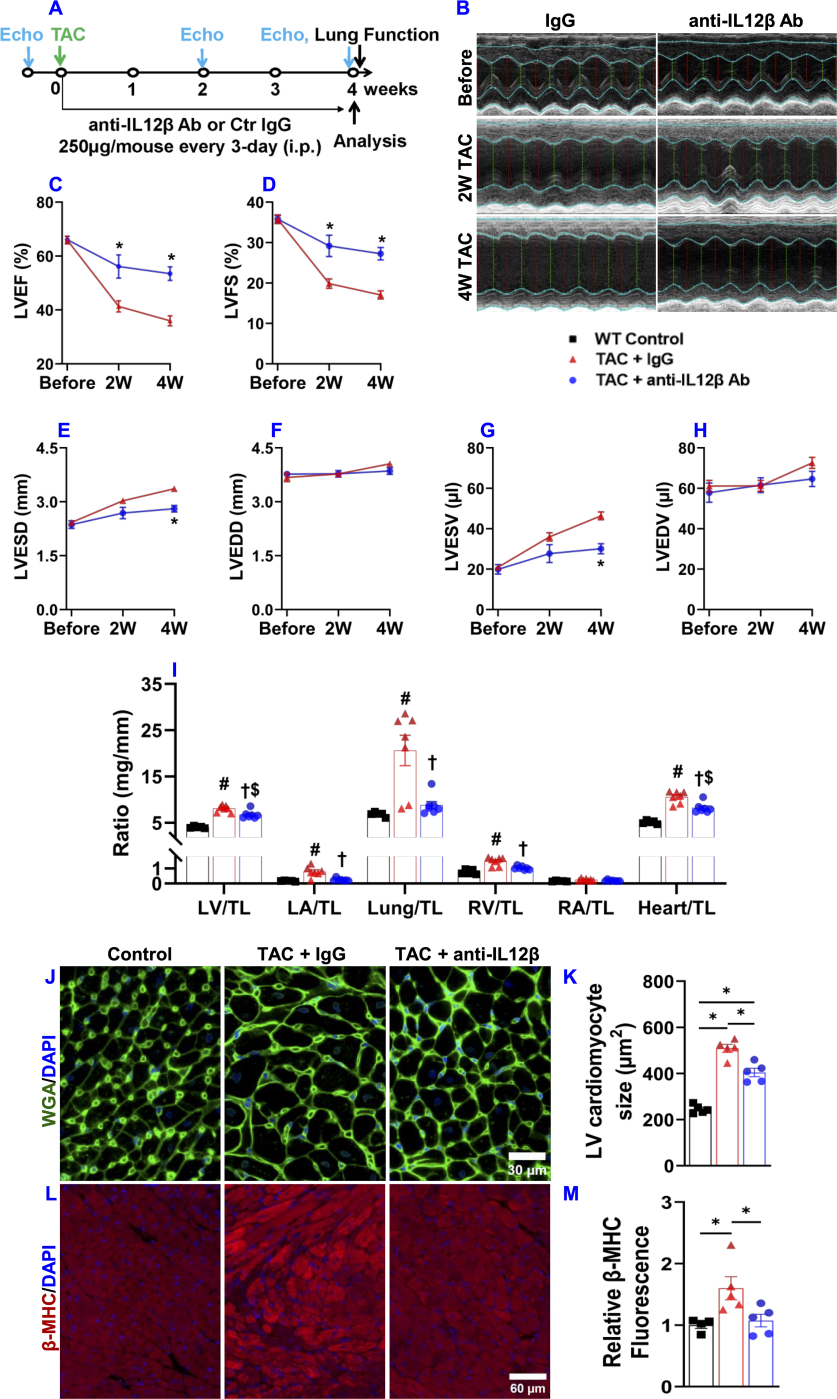
Pharmacological inhibition of IL12β attenuated TAC-induced cardiac dysfunction, LV hypertrophy, increases in lung weight, and RV hypertrophy in mice. **(A)** Schematic diagram of the experimental design. **(B)** Representative M-mode echocardiographic images of the indicated groups. **(C–H)** Quantified data of echocardiographic measurements of LV ejection fraction (LVEF), LV fractional shortening (LVFS), LV end-systolic diameter (LVESD), LV end-diastolic diameter (LVEDD), LV end-systolic volume (LVESV), and LV end-diastolic volume (LVEDV), respectively. **(I)** The ratio of LV weight, left atrial (LA) weight, lung weight, RV weight, right atrial (RA), and total heart weight to tibial length (TL) of the indicated groups. **(J, K)** Representative wheat germ agglutinin (WGA) staining images and quantified data of LV cardiomyocyte cross-sectional area of the indicated groups. **(L, M)** Representative images and quantification of β-MHC expression in LV tissues of the indicated groups. *p<0.05; ^#^p<0.05 IgG-treated TAC mice compared with the control; ^†^p<0.05 anti-IL12β-treated TAC mice compared with IgG-treated TAC mice; ^$^p<0.05 anti-IL12β-treated TAC mice compared with the control; n = 5–7 mice per group.

We investigated the effect of IL12β inhibition by blocking antibody on TAC-induced cardiac inflammation and HF development in wild-type mice using a protocol illustrated in **Figure 1A**. As compared to corresponding sham mice, TAC caused significantly reduced LV ejection fraction and fractional shortening, and significantly increased LV end-systolic diameter in both IgG or anti-IL12β antibody-treated mice, while TAC caused significantly less reduction of LV ejection fraction and fractional shortening in mice treated with anti-IL12β antibody as compared with mice treated with IgG (**Figures 1B–H**).

### 3.2 Pharmacological inhibition of IL12β suppressed TAC-induced LV hypertrophy, increases in lung weight, and RV hypertrophy in mice

IL12β blocking antibody also significantly attenuated the TAC-induced increase of LV weight, left atrial (LA) weight, lung weight, RV weight, and their ratios to tibial length or body weight as compared with mice treated with control IgG (**Figure 1I**, **Supplementary Figure 3, Supplementary Table 2**). We further determined LV cardiomyocyte hypertrophy using FITC-conjugated WGA staining. We found that anti-IL12β antibody treatment significantly decreased TAC-induced cardiomyocyte hypertrophy (**Figures 1J, K**). In addition, IL12β antibody significantly attenuated TAC-induced LV expression of β-myosin heavy chain (β-MHC), a commonly used biomarker of pathological cardiac remodeling and dysfunction (**Figures 1L, M**). However, the expression of β-MHC in the RV tissue was not significantly different among these groups (**Supplementary Figure 4**).

### 3.3 Pharmacological inhibition of IL12β attenuated TAC-induced LV inflammation and fibrosis

**Figure 2:**
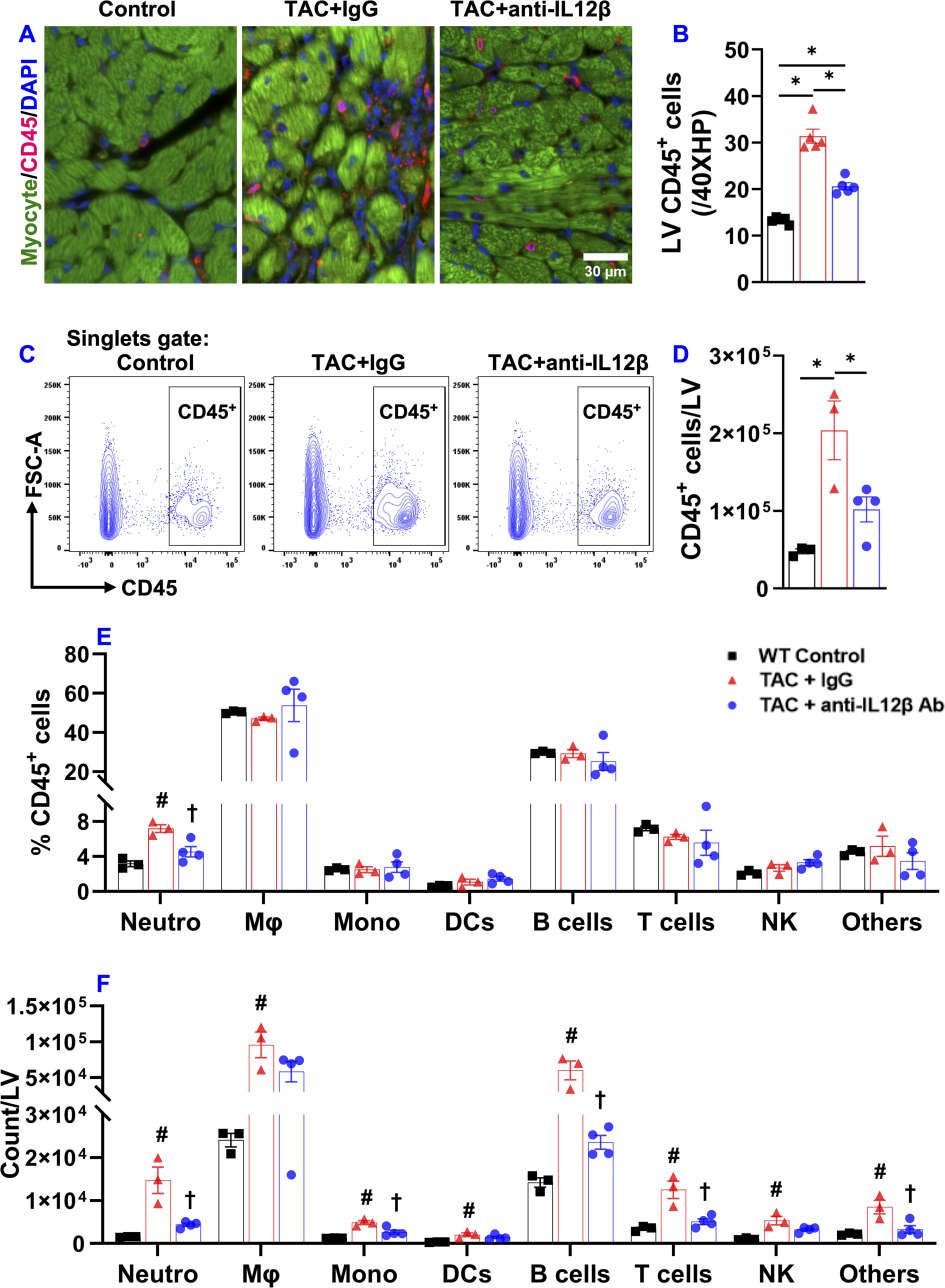
Pharmacological inhibition of IL12β attenuated TAC-induced LV inflammation. **(A, B)** Representative images and quantified data of LV CD45^+^ leukocytes performed by immuno-histological staining. **(C)** Flow cytometry plot for the identification of CD45^+^ leukocytes in the LV. **(D)** Quantified data of the number of CD45^+^ leukocytes per LV. **(E)** The percentage of immune cell subsets within CD45^+^ leukocytes. **(F)** Quantified data of number of different immune cell subsets per LV. *p<0.05; ^#^p<0.05 IgG-treated TAC mice compared with the control; **^†^** p<0.05 anti-IL12β-treated TAC mice compared with IgG-treated TAC mice; Neutro, Neutrophils; Mφ, Macrophage; Mono, Monocytes; DCs, Dendritic Cells; NK, Natural Killer Cells; n = 3–5 mice per group.

**Figure 3:**
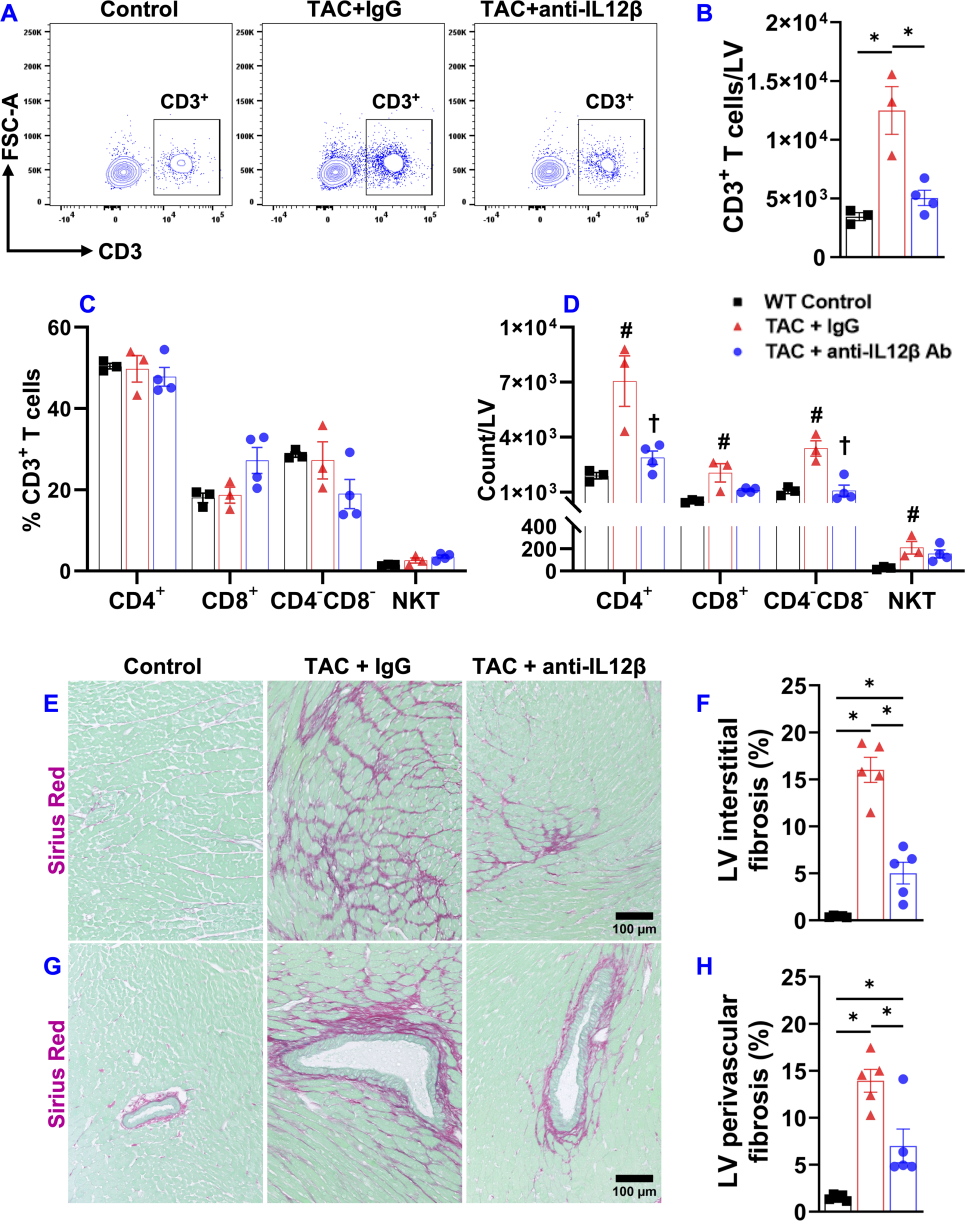
Pharmacological inhibition of IL12β attenuated TAC-induced T cell accumulation in the LV and LV fibrosis. **(A)** Flow cytometry plots for the identification of CD3^+^ T cells in the LV. **(B)** Quantified data of the number of CD3^+^ T cells per LV. **(C)** The percentage of immune cell subsets within CD3^+^ T cells. **(D)** The number of different immune cell subsets per LV. **(E–H)** Representative LV interstitial and perivascular fibrosis and quantified data of fibrosis of the indicated groups, respectively. *p<0.05; ^#^p<0.05 IgG-treated TAC mice compared with the control; **^†^** p<0.05 anti-IL12β-treated TAC mice compared with IgG-treated TAC mice; n = 3–5 mice per group.

Since inflammation often promotes cardiac fibrosis and dysfunction, we further determined the effect of anti-IL12β antibody on TAC-induced LV immune cell infiltration and fibrosis. Histological staining showed that anti-IL12β antibody significantly attenuated TAC-induced LV CD45^+^ leukocyte infiltration (**Figures 2A, B**). We also determined the percentage of different immune cell subsets within CD45^+^ leukocytes and the number of different immune cell subsets based on their expression of immune cell-specific surface markers (**Figures 2C–F**). TAC-induced LV CD45^+^ leukocyte infiltration was significantly attenuated in mice treated with anti-IL12β antibody (**Figures 2C, D**). Although the percentage of different immune cell subsets within CD45^+^ leukocytes except for neutrophils was not significantly changed after the anti-IL12β antibody (**Figure 2E**), the total numbers of several immune cell subsets per LV were significantly increased after TAC in mice treated with IgG (**Figure 2F**). Anti-IL12β antibody significantly attenuated TAC-induced LV infiltration of several immune cell subsets (**Figure 2F**), such as neutrophils, monocytes, B cells, and CD3^+^ T cells (**Figure 2F**). LV CD3^+^ T cells were further grouped as CD4^+^ T cells, CD8^+^ T cells, CD4^-^CD8^-^ T cells, and natural killer T (NKT) cells. TAC caused a significant increase of CD4^+^ T cells, CD8^+^T cells, CD3^+^CD4^-^CD8^-^ T cells, and NKT cells per LV, while anti-IL12β antibody treatment attenuated these changes (**Figures 3A–D**). Since we previously found that T cell activation regulates TAC-induced HF in mice, we also determined the activation status of CD3^+^, CD4^+^, and CD8^+^ T cells based on their expression of CD44 and CD62L. TAC caused a significant increase in the percentage of effector memory CD8^+^ T cells (CD8^+^CD44^+^CD62L^-^) in IgG-treated mice but not in anti-IL12β antibody-treated mice (**Supplementary Figure 5**).

Moreover, histological staining showed that the anti-IL12β antibody significantly reduced TAC-induced LV interstitial and perivascular fibrosis (**Figures 3E–H**).

### 3.4 IL12β blocking antibody reduced HF-induced pulmonary dysfunction, fibrosis, and vascular remodeling in mice

**Figure 4:**
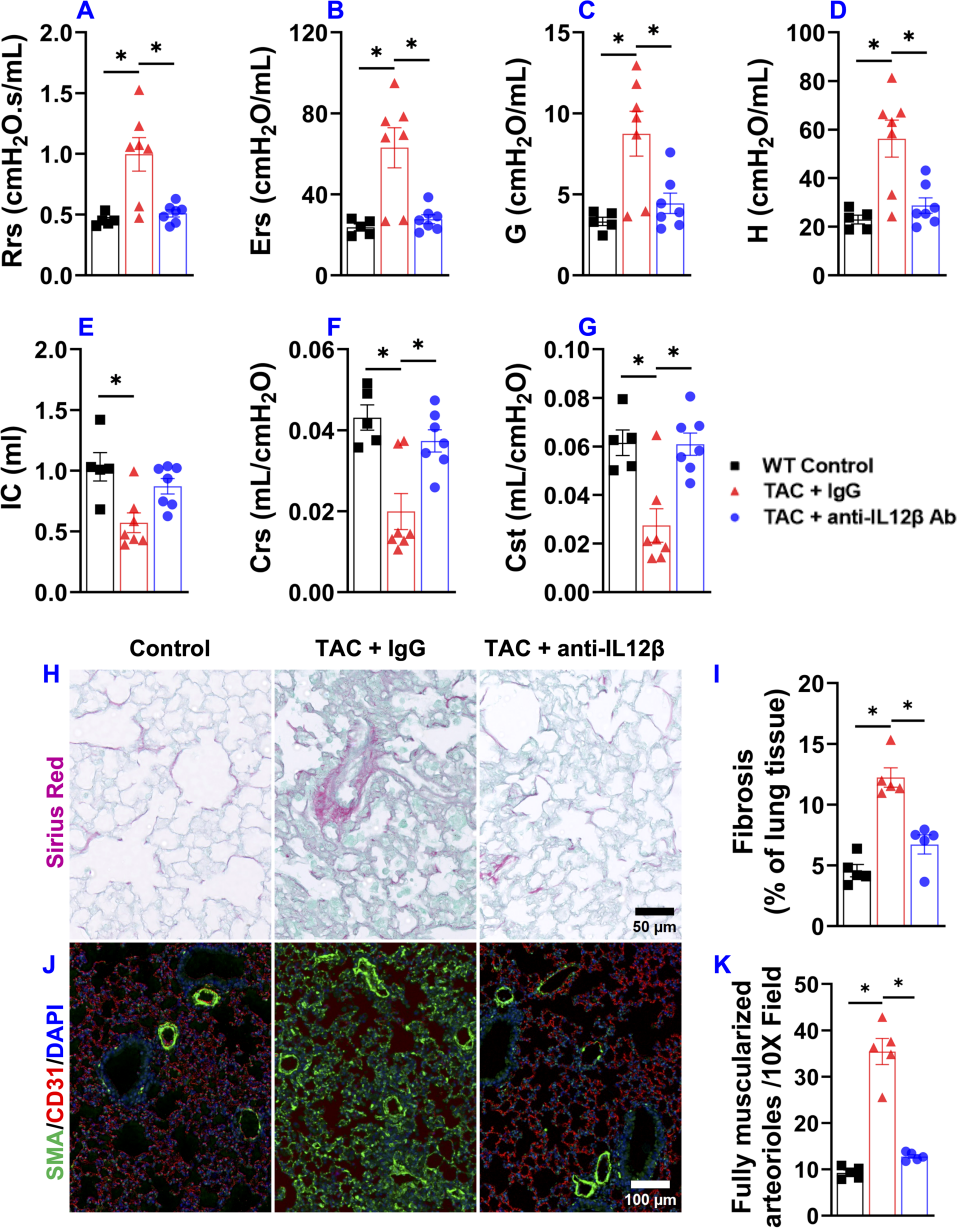
Pharmacological inhibition of IL12β attenuated pulmonary dysfunction and remodeling in wild-type mice. **(A–G)** Quantified data of resistance of respiratory system (Rrs), elastance of respiratory system (Ers), tissue damping **(G)**, tissue elastance **(H)**, inspiratory capacity (IC), compliance of respiratory system (Crs), and quasi-static compliance (Cst) of the indicated groups, respectively. **(H, I)** Representative images and quantified data of lung fibrosis performed by Sirius Red/Fast Green staining. **(J, K)** Representative images and quantified data of lung vessel remodeling performed by immuno-histological staining. *p<0.05; n = 5–7 mice per group.

Since severe LV failure often causes increased lung weight and WHO class II pulmonary hypertension, and since HF-induced lung remodeling also affects the clinical outcome in HF patients, we further determined the effect of anti-IL12β antibody on TAC-induced pulmonary function using SCIREQ’s flexiVent system. As presented in **Figure 4**, TAC caused significant increases in the overall resistance of the respiratory system, elastance of the respiratory system, tissue damping, and tissue elastance (**Figures 4A–D**). TAC also caused a significant decrease in inspiratory capacity, compliance of the respiratory system, and quasi-static compliance (**Figures 4E–G**). Anti-IL12β antibody significantly attenuated TAC-induced pulmonary dysfunction, as the above abnormal changes were normalized by the treatment (**Figures 4A–G**).

Since the biophysical properties such as fibrosis and vessel muscularization could affect pulmonary function, we further determined pulmonary fibrosis and vessel remodeling in TAC mice treated by anti-IL12β antibody or IgG. Interestingly, the anti-IL12β antibody significantly reduced TAC-induced pulmonary fibrosis and vessel muscularization (**Figures 4H–K**). Moreover, TAC also caused massive increases in lung vascular smooth muscle α-actin^+^ cells which was largely abolished by anti-IL12β antibody treatment (**Figure 4J**).

### 3.5 Pharmacological inhibition of IL12β attenuated TAC-induced pulmonary immune cell infiltration

**Figure 5:**
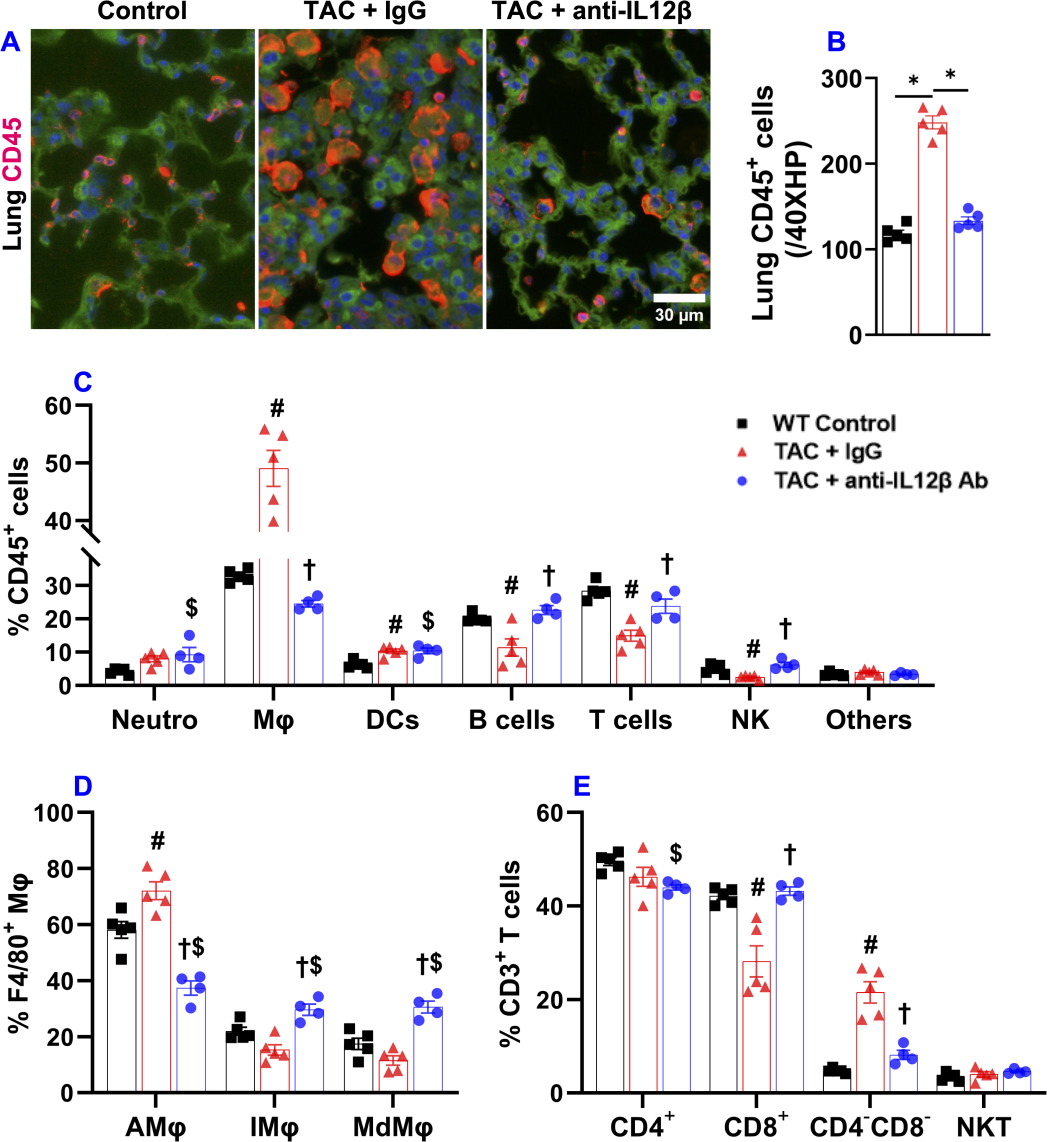
Pharmacological inhibition of IL12β attenuated TAC-induced pulmonary inflammation. **(A, B)** Representative images and quantified data of infiltrated CD45^+^ leukocytes in the lung performed by immuno-histological staining. **(C)** The percentage of immune cell subsets within CD45^+^ leukocytes. **(D)** The percentage of different macrophage subsets within F4/80 macrophages. **(E)** The percentage of immune cell subsets within CD3^+^ T cells. *p<0.05; ^#^p<0.05 IgG-treated TAC mice compared with the control; **^†^** p<0.05 anti-IL12β-treated TAC mice compared with IgG-treated TAC mice; ^$^p<0.05 anti-IL12β-treated TAC mice compared with the control; Mφ, Macrophage; DCs, Dendritic Cells; NK, Natural Killer Cells; AMφ, Alveolar Mφ; IMφ, Interstitial Mφ; MdMφ, Monocyte-derived Mφ; NKT, Natural Killer T Cells; n = 4–5 mice per group.

Our previous studies also demonstrated that chronic HF causes significant lung inflammation as indicated by increased immune cell infiltration and activation, and increased production of pro-inflammatory cytokines by the infiltrated immune cells (2, 23, 24). In addition, modulation of lung inflammatory response is effective in attenuating HF progression (24). Therefore, we sought to determine the effect of anti-IL12β antibody treatment on HF-induced lung inflammation. The pulmonary infiltration of CD45^+^ leukocytes was significantly increased after TAC, while anti-IL12β treatment attenuated the pulmonary infiltration of CD45^+^ leukocytes (**Figures 5A, B**). The percentages of F4/80^+^ Mφ and CD11c^+^ DCs within CD45^+^ leukocytes were significantly increased after TAC, while the percentage of B cells, T cells, and NK cells within CD45^+^ leukocytes were significantly decreased after TAC (**Figure 5C**). The pulmonary F4/80^+^ Mφ were further grouped into alveolar Mφ (AMφ), Ly6C^low^ interstitial Mφ (IMφ), monocyte-derived Ly6C^high^ interstitial Mφ (MdMφ), and CD3^+^ T cells were further grouped into CD4^+^, CD8^+^, CD4^-^CD8^-^, and NKT cells (**Figures 5D, E**).

### 3.6 IL12β blocking antibody significantly attenuated TAC-induced activation of pulmonary alveolar and interstitial macrophages

**Figure 6:**
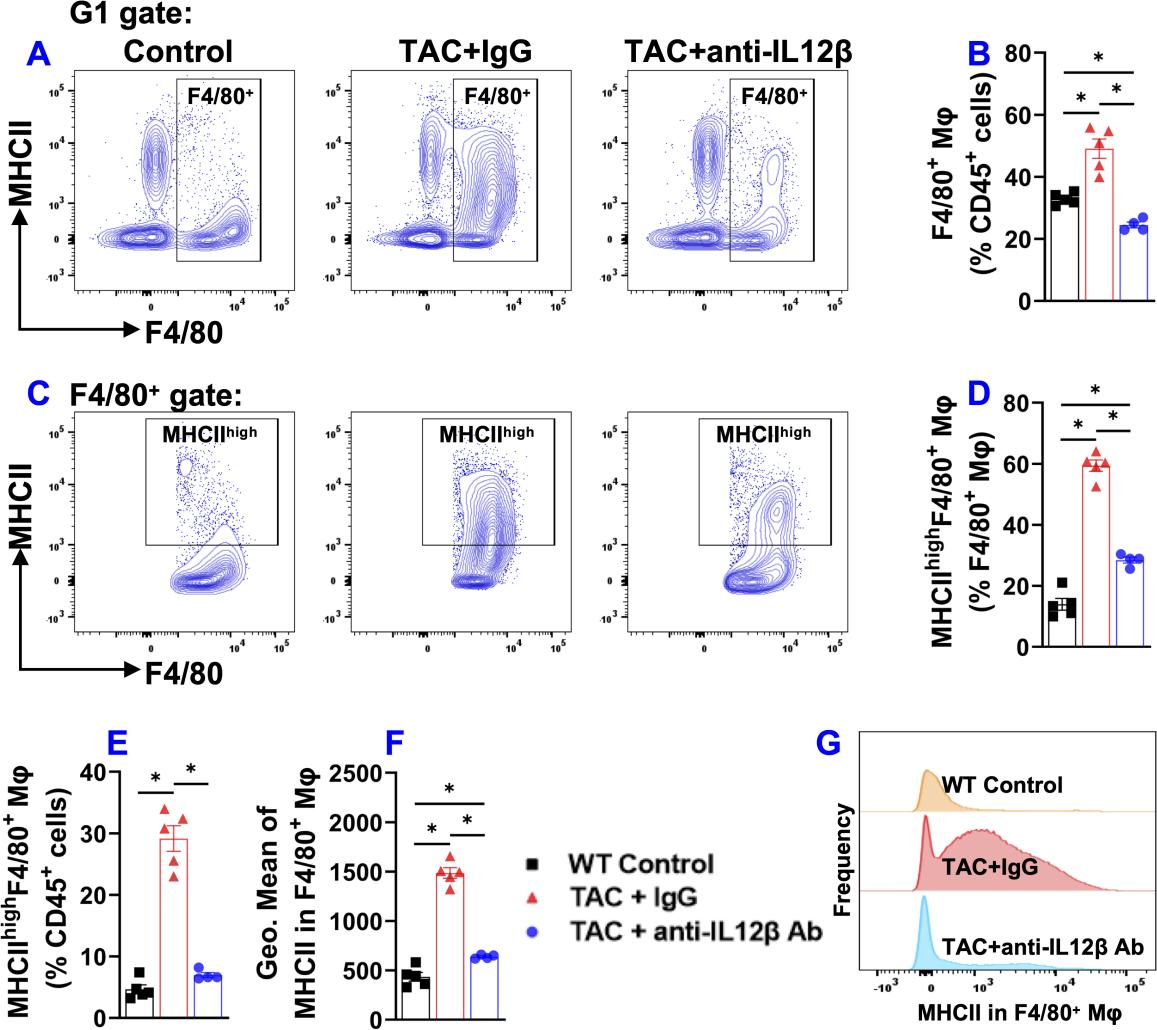
Anti-IL12β antibody treatment attenuated TAC-induced pulmonary F4/80^+^ macrophage accumulation and activation. **(A)** Flow cytometry plots for F4/80^+^ macrophages. **(B)** Quantified data of the percentage of F4/80^+^ cells within CD45^+^ cells. **(C)** Flow cytometry plots for the detection of MHCII expression in F4/80^+^ cells. **(D, E)** Quantified data of the percentage of MHCII^high^F4/80^+^ cells within F4/80^+^ and CD45^+^ cells, respectively. **(F)** Quantified data of mean fluorescent intensity of MHCII in F4/80^+^ cells. **(G)** Representative histograms of MHCII expression in F4/80^+^ cells of the indicated groups. *p<0.05; n = 4–5 mice per group.

Since lung inflammation promotes the transition from LV failure to pulmonary remodeling and RV failure, and since macrophages regulate lung inflammation, we further determined the effect of IL12β blocking antibody on TAC-induced accumulation and activation of pulmonary macrophages (**Figure 6**). Pulmonary F4/80^+^ macrophages, MHCII^high^F4/80^+^ macrophages, and their overall MHCII protein expression in F4/80^+^ macrophages (as indicated by the geometric mean of MHCII) were significantly increased in IgG-treated mice after TAC, while IL12β antibody effectively attenuated TAC-induced increase of the percentage of pulmonary F4/80^+^ macrophages, MHCII^high^F4/80^+^ macrophages, and the average expression of MHCII in these cells (**Figures 6A–F**). Histogram analysis also showed that IL12β antibody treatment effectively attenuated the frequency of MHCII^high^ macrophages within F4/80^+^ cells (**Figure 6G**).

**Figure 7:**
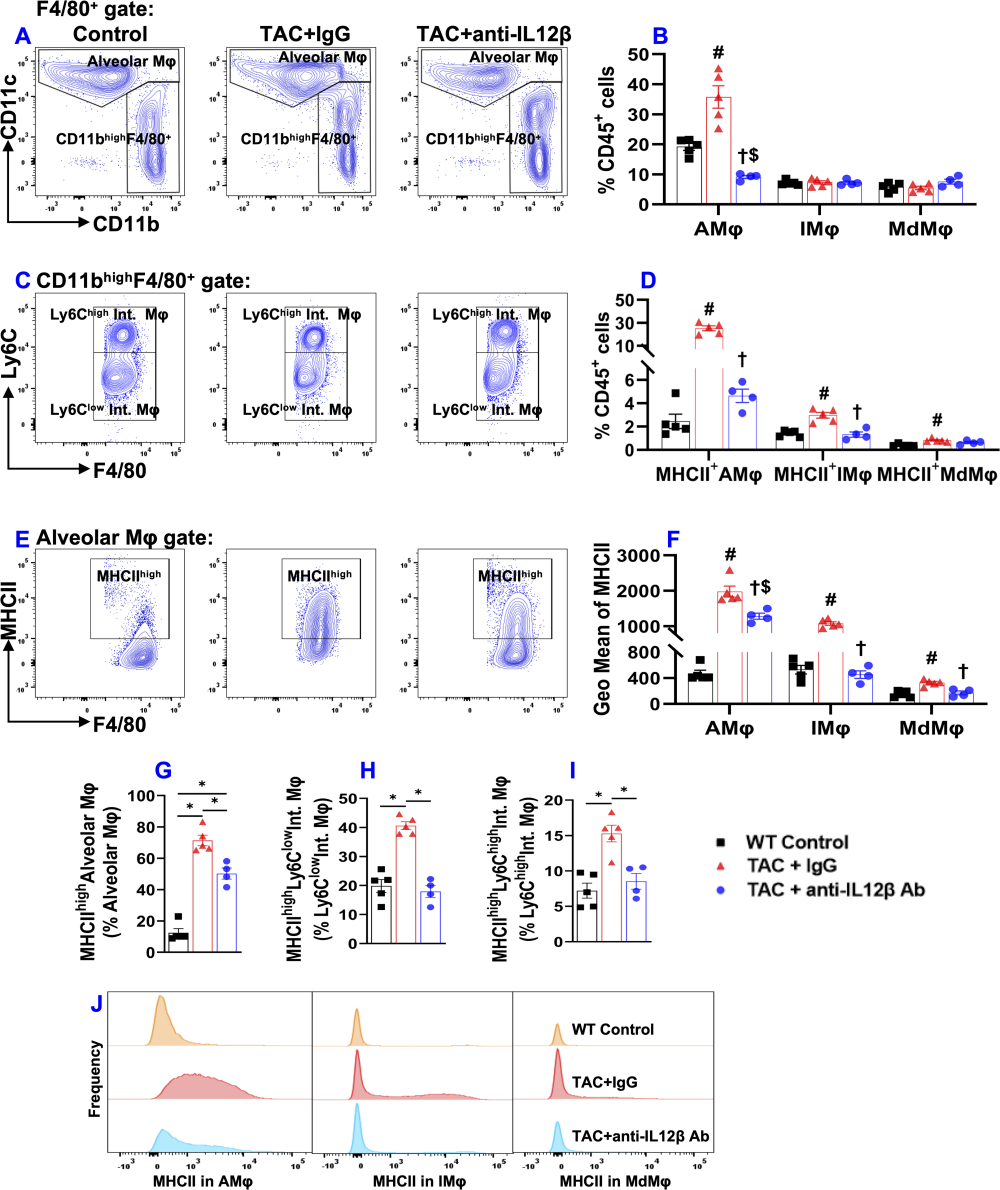
Anti-IL12β antibody treatment attenuated TAC-induced alveolar macrophage accumulation and activation of alveolar and interstitial macrophages. **(A)** Flow cytometry plots of lung alveolar macrophages (AMφ). **(B)** Quantified data of the percentage of AMφ, Ly6C^low^ interstitial macrophages (IMφ), and monocyte-derived Ly6C^high^ interstitial macrophages (MdMφ) within CD45^+^ cells. **(C)** Flow cytometry plots for the identification of IMφ and MdMφ. **(D)** Quantified data of the percentage of MHCII^high^AMφ, MHCII^high^IMφ, and MHCII^high^MdMφ within CD45^+^ leukocytes. **(E)** Flow cytometry plots for the detection of MHCII expression in AMφ. **(F)** Quantified data of mean fluorescent intensity of MHCII in AMφ, IMφ, and MdMφ. **(G-I)** Quantified data of MHCII^high^AMφ, MHCII^high^IMφ, and MHCII^high^MdMφ within AMφ, IMφ, and MdMφ, respectively. **(J)** Representative histograms of MHCII expression in AMφ, IMφ, and MdMφ of the indicated groups. *p<0.05; ^#^p<0.05 IgG-treated TAC mice compared with the control; ^†^p<0.05 anti-IL12β-treated TAC mice compared with IgG-treated TAC mice; ^$^p<0.05 anti-IL12β-treated TAC mice compared with the control; MHCII^high^ (MHCII^+^); n = 4–5 mice per group.

Since pulmonary macrophages are often classified into different macrophage subsets according to their relative expression of CD11c, CD11b, Ly6C, etc., we further determined pulmonary alveolar macrophages (CD11c^high^CD11b^low^F4/80^+^), Ly6C^low^ interstitial macrophages (Ly6C^low^CD11c^low^CD11b^high^F4/80^+^), monocyte-derived Ly6C^high^ interstitial macrophages (Ly6C^high^CD11c^low^CD11b^high^F4/80^+^), and their expression of MHCII (**Figure 7**). As presented in **Figure 7**, the percentage of CD11c^high^CD11b^low^F4/80^+^ alveolar macrophages (AMφ) and the expression of MHCII in AMφ were significantly increased in IgG-treated TAC mice, whereas IL12β antibody significantly attenuated TAC-induced increase of alveolar macrophages and their MHCII expression (**Figures 7A, B, D–G**). TAC caused no significant changes in interstitial Ly6C^low^CD11c^low^CD11b^high^F4/80^+^ macrophages (IMφ) and monocyte-derived Ly6C^high^CD11c^low^CD11b^high^F4/80^+^ macrophages (MdMφ) in mice with or without IL12β antibody treatment (**Figures 7B, C**). Interestingly, MHCII expression in pulmonary IMφ and MdMφ were significantly increased in IgG-treated TAC mice, whereas anti-IL12β antibody significantly attenuated MHCII expression in these immune cells (**Figures 7D, F, H, I**). Histogram analysis also showed that IL12β antibody treatment effectively attenuated the frequency of MHCII^high^ macrophages within macrophage subsets (**Figure 7J**).

### 3.7 IL12β blocking antibody significantly reduced TAC-induced activation of pulmonary CD11c^+^ dendritic cells

**Figure 8:**
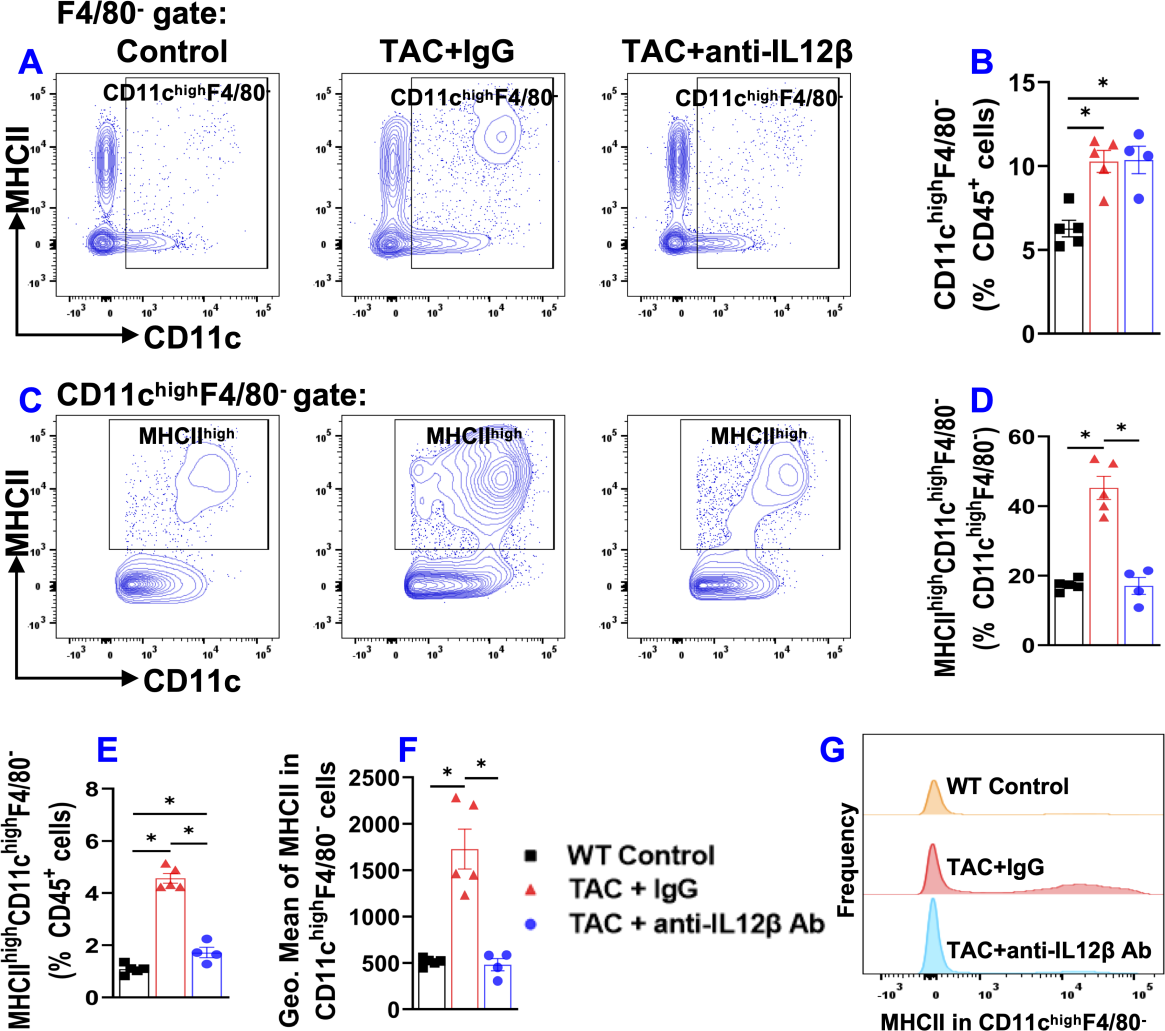
Anti-IL12β antibody treatment attenuated TAC-induced activation of pulmonary dendritic cells (CD11c^high^F4/80^-^). **(A)** Flow cytometry plots of lung CD11c^high^F4/80^-^ cells. **(B)** Quantified data of the percentage of CD11c^high^F4/80^-^ cells within CD45^+^ cells. **(C)** Flow cytometry plots for the detection of MHCII expression in CD11c^high^F4/80^-^ cells. **(D, E)** Quantified data of the percentage of MHCII^high^CD11c^high^F4/80^-^ cells within CD11c^high^F4/80^-^ cells and CD45^+^ cells, respectively. **(F)** Quantified data of mean fluorescent intensity of MHCII in CD11c^high^F4/80^-^ cells. **(G)** Representative histograms of MHCII expression in CD11c^high^F4/80^-^ cells of the indicated groups. *p<0.05; n = 4–5 mice per group.

Since CD11c^+^ antigen-presenting cells play an important role in regulating tissue inflammation and/or HF development (21), we measured pulmonary CD11c^+^ dendritic cells (**Figure 8**). Since pulmonary alveolar macrophages also express CD11c, pulmonary dendritic cells were gated as F4/80^-^CD11c^+^ cells as shown in the gating strategy (**Supplementary Figure 1**). TAC caused a significant pulmonary accumulation of CD11c^+^ dendritic cells in both anti-IL12β antibody and IgG-treated mice as compared to the control mice (**Figures 8A, B**). We also determined the percentage of MHCII^high^CD11c^+^ dendritic cells within CD11c^+^ dendritic cells or within CD45^+^ leukocytes (**Figures 8C–E**). The percentage of MHCII^high^CD11c^+^ dendritic cells within CD11c^+^ dendritic cells and CD45^+^ leukocytes was significantly increased in IgG-treated TAC mice and significantly reduced in mice treated with anti-IL12β antibody (**Figures 8D, E**). In addition, the anti-IL12β antibody also significantly attenuated the TAC-induced increase of the average expression of MHCII protein in CD11c^+^ dendritic cells, as determined by the GEO mean (**Figure 8F**). Moreover, the histogram also showed that the frequency of MHCII^high^ dendritic cells was significantly increased in IgG-treated TAC mice, while this change was completely abolished with anti-IL12β treatment (**Figure 8G**).

### 3.8 IL12β blocking antibody effectively attenuated TAC-induced pulmonary T cell activation

**Figure 9:**
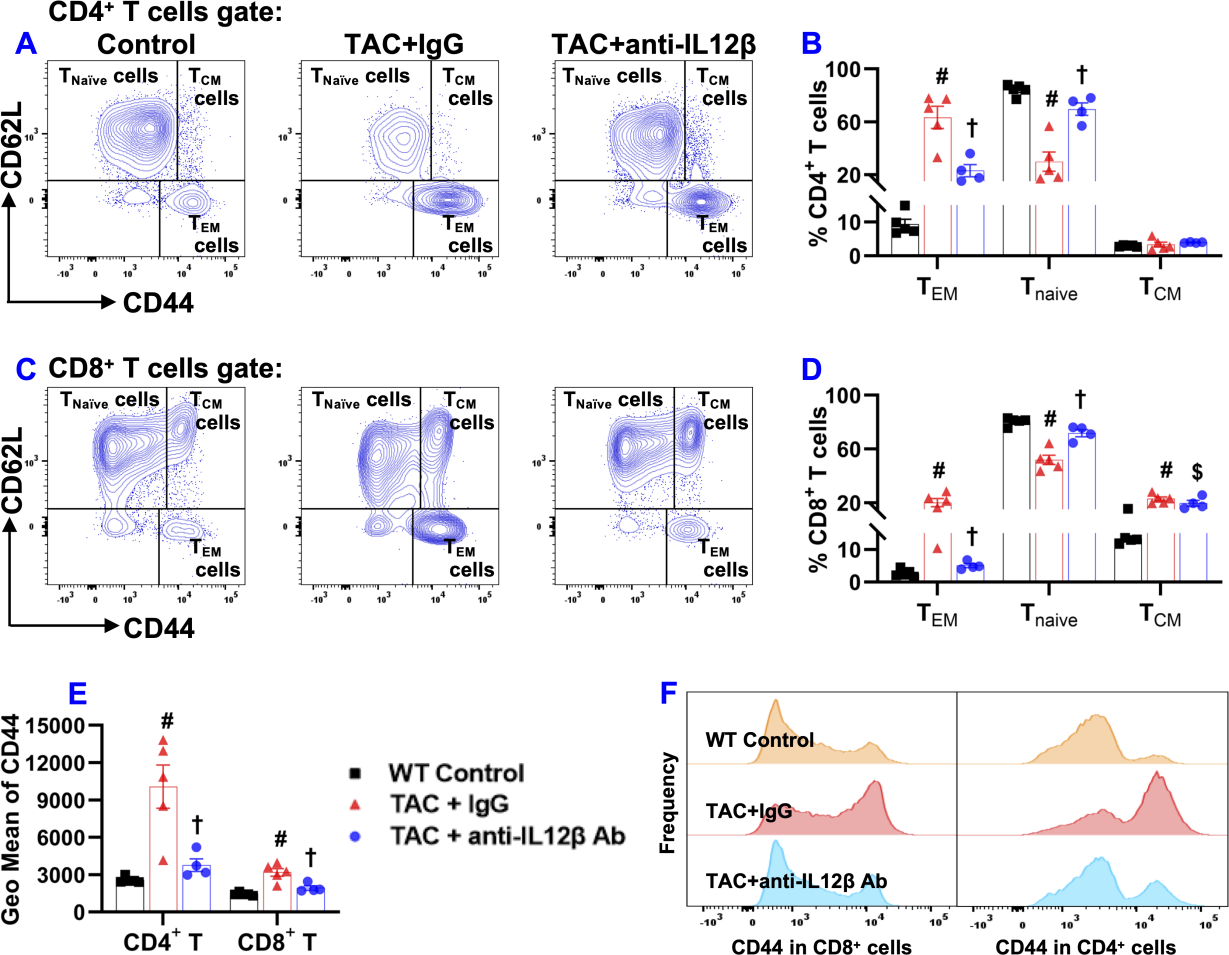
Anti-IL12β antibody treatment attenuated TAC-induced pulmonary T cell activation. **(A)** Flow cytometry plots for the detection of the activation status of CD4^+^ T cells. **(B)** Quantified data of the percentage of CD44^+^CD62L^-^CD4^+^ effector memory (T_EM_) cells, CD44^-^CD62L^+^CD4^+^ naïve cells (T_naïve_), and CD44^+^CD62L^+^CD4^+^ central memory (T_CM_) cells within CD4^+^ T cells. **(C)** Flow cytometry plots for the detection of the activation status of CD8^+^ T cells. **(D)** Quantified data of the percentage of CD44^+^CD62L^-^CD8^+^ T_EM_ cells, CD44^-^CD62L^+^CD8^+^ T_naïve_ cells, and CD44^+^CD62L^+^CD8^+^ T_CM_ cells within CD8^+^ T cells. **(E)** Quantified data of mean fluorescent intensity of CD44 in CD4^+^ T and CD8^+^ T cells. **(F)** Representative histograms of CD44 expression in CD4^+^ T and CD8^+^ T cells of the indicated groups. ^#^p<0.05 IgG-treated TAC mice compared with the control; **^†^** p<0.05 anti-IL12β-treated TAC mice compared with IgG-treated TAC mice; ^$^p<0.05 anti-IL12β-treated TAC mice compared with the control; n = 4–5 mice per group.

In the context of the important role of T cells in cardiopulmonary inflammation (11), we further determined pulmonary T cell accumulation and activation. Due to the significant increase of pulmonary macrophages, the relative percentage of pulmonary CD3^+^, CD4^+^, and CD8^+^ T cells within CD45^+^ leukocytes decreased significantly after TAC in IgG-treated mice but not in anti-IL12β antibody-treated mice (**Supplementary Figure 6**). The pulmonary effector memory T cell subset (CD44^+^CD62L^-^), naïve T cell subset (CD44^-^CD62L^+^), the central memory T cells (CD44^+^CD62L^+^), and exhausted T cell subset (CD44^-^CD62L^-^) of the major T cell subsets are presented in **Figure 9** and **Supplementary Figure 6**. Overall, these data showed that the anti-IL12β antibody significantly attenuated the TAC-induced increase of pulmonary effector memory T cell subsets and the decrease of naïve T cell subset of CD3^+^ T cells, CD4^+^ T cells, and CD8^+^ T cells.

TAC caused a significant increase in CD3^+^ effector memory T cells (CD44^+^CD62L^-^CD3^+^) and a significant decrease in CD3^+^ naïve T cells (CD44^-^CD62L^+^CD3^+^) in IgG-treated mice, while the above changes were completely abolished in the mice treated with anti-IL12β antibody (**Supplementary Figure 6**). The percentage of CD3^+^ central memory T cells (CD44^+^CD62L^+^CD3^+^) was significantly increased in anti-IL12β antibody-treated mice as compared to the control mice (**Supplementary Figure 6**). In addition, anti-IL12β treatment significantly decreased the TAC-induced increase of CD44 (a protein that facilitates immune cells homing to the injured or infected tissue) expression in CD3^+^ T cells (**Supplementary Figure 6**). Moreover, the histogram also showed that the frequency of CD44^+^CD3^+^ T cells was significantly increased in IgG-treated TAC mice, while this effect was abolished with anti-IL12β treatment (**Supplementary Figure 6**).

CD3^+^ T cells were further grouped into CD4^+^ and CD8^+^ T cells (**Supplementary Figure 6**). As expected, TAC caused a significant increase in effector memory CD4^+^ T cells (CD44^+^CD62L^-^CD4^+^) and a significant decrease in naïve CD4^+^ T cells (CD44^-^CD62L^+^CD4^+^) in IgG-treated mice, while these changes were abolished entirely in the anti-IL12β antibody-treated mice (**Figures 9A, B**). The percentage of central memory CD4^+^ T cells (CD44^+^CD62L^+^CD4^+^) was unchanged between different groups (**Figures 9A, B**). Similar to CD4^+^ T cells, TAC caused a significant increase in effector memory CD8^+^ T cells (CD44^+^CD62L^-^CD8^+^), and a significant decrease in naïve CD8^+^ T cells (CD44^-^CD62L^+^CD8^+^) in IgG-treated mice, and these changes were abolished entirely in the mice treated with anti-IL12β antibody (**Figures 9C, D**). The percentage of central memory CD8^+^ T cells (CD44^+^CD62L^+^CD8^+^) significantly increased in both groups of TAC mice compared to the control mice (**Figures 9C, D**). In addition, the average expression of CD44 protein in CD4^+^ T and CD8^+^ T cells, as determined by GEO mean, was significantly increased after TAC in IgG-treated mice but not in anti-IL12β antibody-treated mice (**Figure 9E**). Furthermore, the histogram showed that the frequency distribution of CD44 in CD4^+^ T and CD8^+^ T cells was significantly increased in the TAC mice treated with IgG, while anti-IL12β antibody treatment abolished this change (**Figure 9F**).

### 3.9 Anti-IL12β antibody significantly attenuated TAC-induced pro-inflammatory cytokine production by pulmonary T cells and macrophages

**Figure 10:**
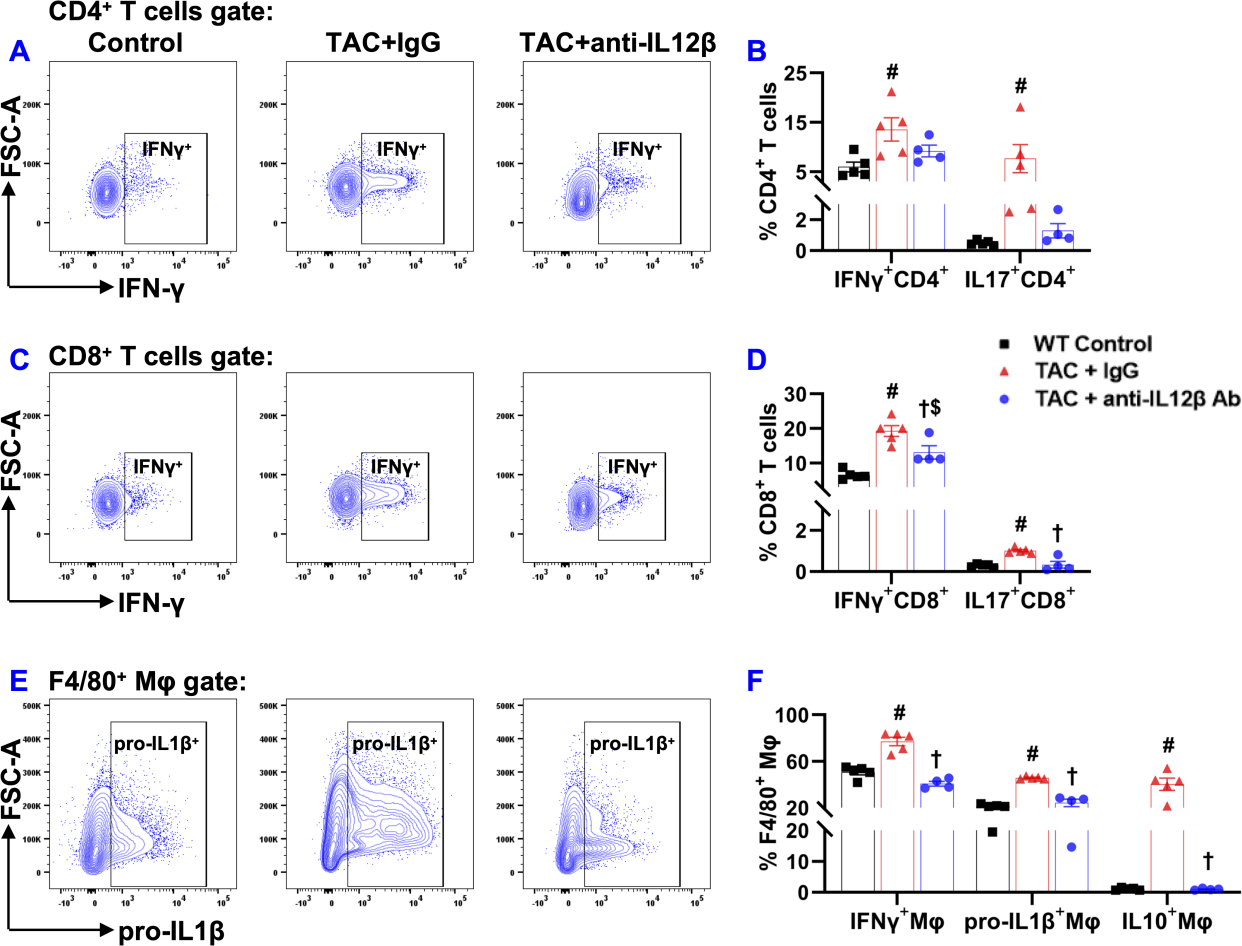
Anti-IL12β antibody treatment attenuated TAC-induced pro-inflammatory cytokine production by pulmonary T cells and macrophages. **(A)** Flow cytometry plots for the detection of IFNγ production by CD4^+^ T cells. **(B)** Quantified data of IFNγ^+^ and IL17^+^ cells within CD4^+^ cells. **(C)** Flow cytometry plots for the detection of IFNγ production by CD8^+^ T cells. **(D)** Quantified data of IFNγ^+^ and IL17^+^ cells within CD8^+^ cells. **(E)** Flow cytometry plots for the detection of pro-IL1β production by F4/80^+^ macrophages. **(F)** Quantified data of IFNγ^+^, pro-IL1β^+^, and IL10^+^ cells within F4/80^+^ macrophages. ^#^p<0.05 IgG-treated TAC mice compared with the control; ^†^p<0.05 anti-IL12β-treated TAC mice compared with IgG-treated TAC mice; ^$^p<0.05 anti-IL12β-treated TAC mice compared with the control; n = 4–5 per group.

Since IL12 and IL23 regulate inflammation by promoting the production of Interferon-γ (IFNγ) and IL17 by other immune cells, we further determined cytokine production by CD4^+^ T cells, CD8^+^ T cells, and macrophages by stimulating the cells with a cell stimulation cocktail and protein transport inhibitor cocktail at 37°C for 2 hours. IFNγ production by CD4^+^ T cells was significantly increased in IgG-treated TAC mice but not in anti-IL12β antibody-treated TAC mice (**Figures 10A, B**). Moreover, IFNγ production by CD8^+^ T cells was also significantly increased in both groups of TAC mice, while anti-IL12β treatment drastically decreased IFNγ production by CD8^+^ T cells (**Figures 10C, D**). The percentages of IL17^+^CD4^+^ T cells and IL17^+^CD8^+^ T cells were significantly increased after TAC in IgG-treated mice, while these changes were completely abolished with anti-IL12β treatment (**Figures 10B, D**).

The percentages of pro-IL1β^+^ and IFNγ^+^ cells within F4/80^+^ macrophages were significantly increased in IgG-treated TAC mice, while anti-IL12β antibody treatment completely abolished TAC-induced increases in pro-IL1β and IFNγ production by macrophages (**Figures 10E, F**). The percentages of IL10^+^ cells within F4/80^+^ macrophages were significantly increased in IgG-treated TAC mice, while anti-IL12β antibody treatment completely abolished TAC-induced increase in IL10 production by macrophages (**Figure 10F**).

## 4 Discussion

**Figure 11:**
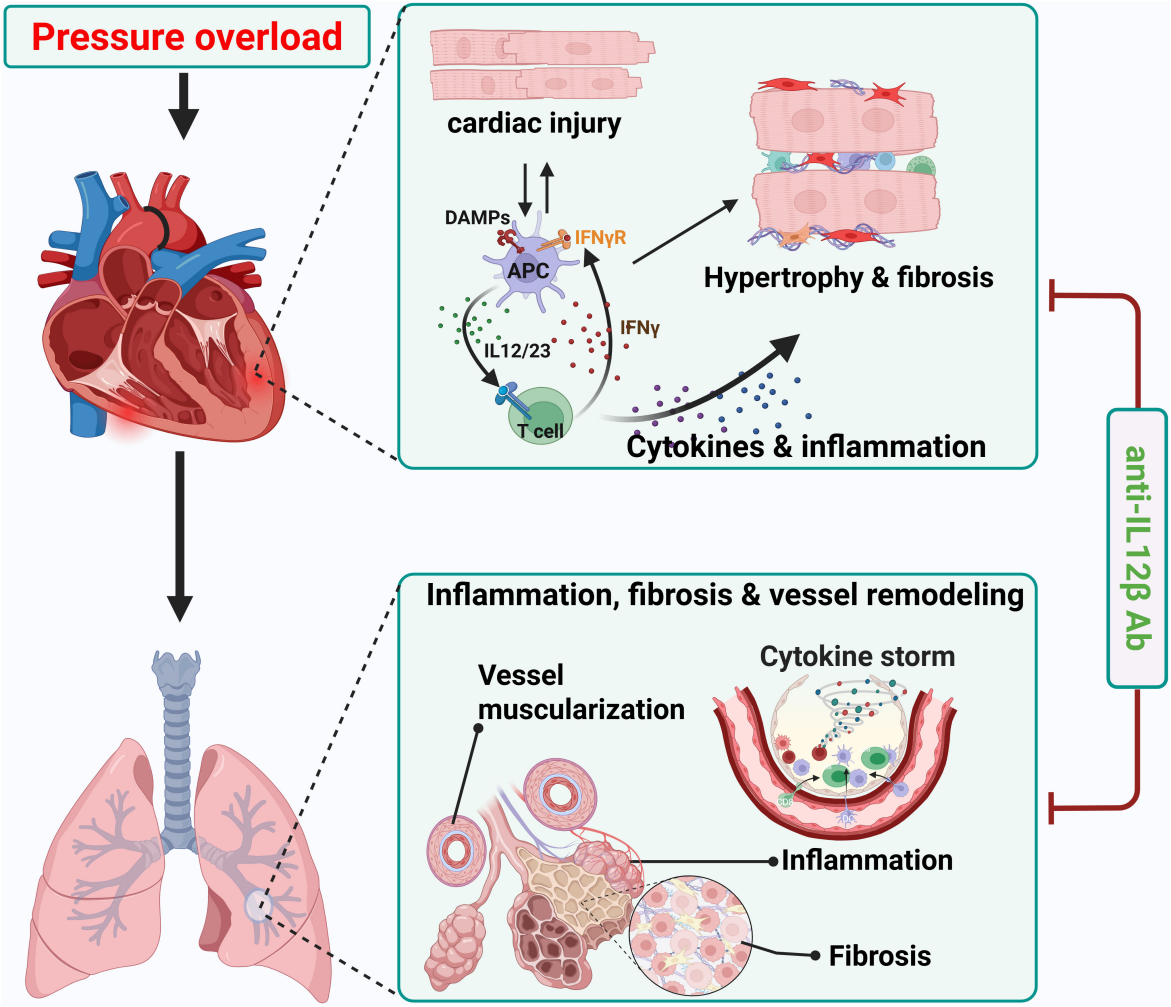
Schematic diagram showing effect of anti-IL12β antibody treatment on TAC-induced heart failure development and progression. Neutralizing IL12β using anti-IL12β antibody attenuates TAC-induced LV inflammation, hypertrophy, dysfunction, and consequent pulmonary inflammation, remodeling, and right ventricular hypertrophy. DAMPs, Damage-associated molecular patterns; APC, Antigen presenting cell.

In the current study, we determined the effect of pharmacological inhibition of IL12β on pressure overload-induced HF development and progression. First, we found that IL12β blocking antibody attenuated TAC-induced LV hypertrophy, fibrosis, dysfunction, and consequent lung dysfunction and remodeling. Second, IL12β blocking antibody significantly reduced TAC-induced LV accumulation of inflammatory immune cell subsets such as macrophages, dendritic cells, B cells, T cells, and activation of CD8^+^ T cells. Third, IL12β blocking antibody attenuated TAC-induced pulmonary immune cell infiltration, the accumulation and activation of F4/80^+^ macrophages, and the major lung macrophage subsets such as alveolar macrophage and monocyte-derived Ly6C^high^ interstitial macrophage. Fourth, IL12β blocking antibody significantly decreased TAC-induced activation of pulmonary CD11c^+^ dendritic cells, CD4^+^ T cells, and CD8^+^ T cells. Fifth, we found that IL12β blocking antibody significantly attenuated the TAC-induced increase of pro-inflammatory cytokine IL17 and IFNγ production by CD4 and/or CD8 T cells, as well as pro-IL1β and IFNγ production by macrophages. Overall, these findings indicate that inhibition of IL12β effectively reduces TAC-induced HF development and progression from LV failure by suppressing multiple cardiopulmonary immune cell infiltration and their production of proinflammatory cytokines such as IFNγ, IL1β, and IL17 (**Figure 11**).

One of the major findings of this study is that inhibition of IL12β significantly attenuated TAC-induced cardiac inflammation and dysfunction, evidenced by the following changes. First, inhibition of IL12β significantly attenuated the TAC-induced decrease of LV ejection fraction, LV fractional shortening, and increase of LV end-systolic diameter and volume. In addition, IL12β blocking antibody significantly ameliorated TAC-induced LV immune cell infiltration, hypertrophy, and fibrosis. These findings of significant cardiac dysfunction, LV hypertrophy, inflammation, and fibrosis in IgG-treated mice after TAC are consistent with the notion that inflammation plays an important role in pressure overload-induced HF development. We and others have previously demonstrated the important role of immune cells such as CD4^+^ T cells (19), CD8^+^ T cells (20), macrophages (22), CD11c^+^ dendritic cells (21), and NK1.1^+^ cells (9) in cardiac inflammation and dysfunction. The current findings that pharmacological inhibition of IL12β significantly attenuates TAC-induced HF development not only confirm the crucial role of inflammation in HF development (2, 11) but also highlight the important role of IL12β in promoting cardiac inflammation and HF development.

Our data also indicate that pharmacological inhibition of IL12β significantly attenuated the TAC-induced increase in lung weight, RV weight, and their ratio to tibial length or body weight, implying that IL12β not only plays a role in pressure overload-induced HF development but also in HF progression. The role of IL12β in TAC-induced HF progression is also confirmed by the findings that inhibition of IL12β ameliorated TAC-induced pulmonary inflammation, dysfunction, fibrosis, and vascular muscularization. The TAC-induced lung dysfunction is consistent with our previous finding that HF is associated with a dramatic increase in lung airway resistance and a significant decrease in lung compliance (4). The HF-induced increase in lung immune cell infiltration, fibrosis, and vessel muscularization could contribute to the lung dysfunction observed in these experimental animals by changing the physical properties of the key components of lung tissues. These changes in lung function could explain the decreased exercise capacity, shortness of breath, poor gas exchange capacity, and decreased arterial oxygen saturation in HF patients. Since LV failure could lead to pulmonary inflammation, remodeling, and RV hypertrophy, the reduced lung inflammation, remodeling, and RV hypertrophy in anti-IL12β antibody-treated mice after TAC could be due to both the improved cardiac function in these mice and the reduced lung inflammation. Nevertheless, since lung inflammation can promote lung remodeling and RV hypertrophy without affecting LV function in mice with pre-existing HF (24) and since IL12β can regulate the inflammatory response, pharmacological inhibition of IL12β may directly reduce lung inflammation and remodeling in HF mice. Inflammation not only plays a role in HF development but also plays a crucial role in HF progression (2, 11, 24). Our previous studies demonstrated that HF is associated with a massive accumulation and activation of macrophages, CD11c^+^ dendritic cells, and activation of T cells in the lungs, while modulation of inflammatory response was effective in attenuating chronic HF-induced pulmonary inflammation, remodeling, and RV hypertrophy in the mice with pre-existing HF (10, 23, 24). For example, enhancing lung inflammation by exposing mice to pm2.5 exacerbated HF-induced lung inflammation and remodeling in mice with pre-existing HF (24). In fact, endogenous induction of regulatory T cells (23), and inhibition of IL1β (10) attenuated HF progression in mice with pre-existing HF. Our current findings of a significant increase in lung inflammation, remodeling, and RV hypertrophy that is attenuated with anti-IL12β antibody in WT mice after TAC are consistent with the notion that lung inflammation and remodeling play a critical role in pressure overload-induced HF progression (11, 21, 23).

Another interesting finding of this study is that TAC caused significant accumulation and activation of macrophages, and dendritic cells, while anti-IL12β treatment largely abolished these changes. Since macrophages and dendritic cells are the major producers of IL12β, a reduced inflammatory environment due to the neutralization of IL12β may impact the expression of MHCII in macrophages and dendritic cells. The increase in MHCII expression in lung macrophages and dendritic cells increases their antigen-presenting capacity and promotes lung inflammation. Moreover, inhibition of IL1β signaling also significantly attenuated TAC-induced HF development and progression, as well as reduced cardiac and pulmonary pro-IL1β production (10). Thus, the current study suggests that the cardiac protective effect of anti-IL12β antibody may be partially mediated through reduced IL1β production by macrophages.

Our data also indicate that anti-IL12β antibody significantly attenuated TAC-induced pulmonary accumulation of activated CD44^+^CD62L^-^CD3^+^ T cells, CD44^+^CD62L^-^CD4^+^ T cells, and CD44^+^CD62L^-^CD8^+^ T cells. IL12β inhibition not only decreased pulmonary accumulation and activation of immune cells but also decreased the pro-inflammatory IFNγ and IL17 production by CD4^+^ T cells and/or CD8^+^ T cells. The decreased pulmonary T cell accumulation, activation, and reduced pro-inflammatory cytokine production by T cells might be an important mechanism of reduced lung inflammation and remodeling with subsequent improvement in lung function with anti-IL12β antibody treatment. Since IL12 promotes T cell IFNγ production, while IL23 promotes IL17 production by T cells, the reduced IFNγ and IL17 production in lung T cells in mice after anti-IL12β antibody is likely an outcome of reduced IL12 and IL23 signaling, respectively. Since IFNγ also promotes IL12 and IL1β production by macrophages and dendritic cells, the reduced cardiopulmonary inflammation in our current study is likely a collective effect of reduced crosstalk among T cells, macrophages, and other major immune cells that infiltrated the affected tissues.

This study has several limitations. First, pharmacological inhibition of IL12β attenuated pressure overload-induced cardiac inflammation, cardiomyocyte hypertrophy, and fibrosis. Since each of these above factors could independently lead to HF development, our study could not fully identify the relative role of each of the above factors. Second, HF alone can promote lung inflammation, remodeling, and RV hypertrophy, therefore, we cannot definitively define the mechanism(s) of IL12β inhibition on HF progression, whether direct and/or indirectly improving RV and lung inflammation. Nevertheless, this study still highlights the crucial role of IL12β inhibition on pressure overload-induced HF development and progression. Third, we only used female mice in this study. Female mice are generally resistant to TAC-induced HF development and progression. Therefore, we anticipate that IL12β inhibition would have similar effects on male mice as well. Fourth, since cardiac inflammation and dysfunction don’t occur in normal heart or control conditions, and since our previous published and unpublished studies consistently showed that blocking antibodies or IgG don’t have detectable effect on cardiac inflammation and function, sham mice were not treated with the blocking antibody in the present study. Fifth, our study is performed in well-controlled healthy young mice for a relatively short period. Since HF generally occurs in unhealthy old patients often suffered from various stresses or chronic diseases, unfriendly environmental conditions (such as air pollution) or pathogens, the findings from the well-controlled study may be different to the complicated clinical conditions. Thus, since immune cells exert important role in controlling lung infection, the potential risk of IL12β antibody (such as Ustekinumab) may increase infection, particular lung infection (55), although anti-IL12β antibody has been generally well tolerated by patients in clinical trials, with most adverse events being mild in severity (56, 57). Nevertheless, without a well-controlled clinical trial, the overall impact of anti-IL12β antibody on cardiopulmonary inflammation and HF development in clinical conditions is still unknown. Moreover, as the main purpose of our study is to determine whether anti-IL12β antibody can block TAC-induced cardiopulmonary inflammation and dysfunction, we only studied a single dose according to the previous reports. Finally, due to our focus is limited to systolic overload induced cardiopulmonary inflammation and dysfunction, the impact of anti-IL12β antibody on other system was not investigated in our study. However, studies demonstrated that inhibition of IL12β can effectively attenuate both IL12/IFNγ and IL23/IL17 axes (25–29), IL12β antibody can attenuate inflammatory conditions associated with IL12/IFNγ and IL23/IL17 axes.

In summary, our data demonstrate that IL12β blocking antibody effectively suppresses pressure overload-induced cardiac dysfunction, hypertrophy, inflammation, fibrosis, and the consequent pulmonary inflammation and dysfunction, as well as RV hypertrophy. These findings highlight the important role of pharmacological inhibition of IL12β in treating systolic overload-induced HF development and progression. Therefore, pharmacological inhibition of IL12β, which is currently used to treat patients with certain autoimmune diseases, may offer an effective therapeutic strategy for treating or halting HF development and progression.

## Data Availability

The original contributions presented in the study are included in the article/**Supplementary Material**. Further inquiries can be directed to the corresponding author.
