# Supplementary material for: Pharmacological inhibition of IL12β is effective in treating pressure overload-induced cardiac inflammation and heart failure
Source: Front Immunol. 2025 Aug 15;16:1624940. doi: 10.3389/fimmu.2025.1624940 (PMC12395052; doi:10.3389/fimmu.2025.1624940)
Supplement: Supplementary file 1 [file DataSheet1.docx]

**Supplementary Materials:**

**Pharmacological inhibition of IL12β is effective in treating pressure overload-induced cardiac inflammation and heart failure**

Umesh Bhattarai^1^, Xiaochen He^1^, Ziru Niu^1^, Lihong Pan^1^, Dongzhi Wang^1^, Hao Wang^1^, Heng Zeng^2^, Jian-Xiong Chen^2^, Joshua S. Speed^1^, John S. Clemmer^1^, Yingjie Chen^1*^

^1^Department of Physiology and Biophysics, School of Medicine, University of Mississippi Medical Center, Jackson, MS, United States

^2^Department of Pharmacology and Toxicology, School of Medicine, University of Mississippi Medical Center, Jackson, MS, United States

***Correspondence**

Yingjie Chen, Ph.D.

Professor

Department of Physiology and Biophysics,

University of Mississippi Medical Center,

2500 North State Street,

Jackson, MS, 39216

Office: 601-815-3986

Email: ychen2@umc.edu

**Supplementary Table 1:** Antibodies used for flow cytometry analyses

| **Antibody** | **Conjugate** | **Clone** | **Vendor** | **Catalog #** |
| --- | --- | --- | --- | --- |
| **CD3e** | BUV737 | 145-2C11 | BD Biosciences | 612771 |
| **CD4** | BUV496 | GK1.5 | BD Biosciences | 612952 |
| **CD8α** | BB790 | 53-6.7 | BD Biosciences | 624296 |
| **CD11b** | BV650 | M1/70 | Biolegend | 101259 |
| **CD11c** | BV711 | N418 | Biolegend | 117349 |
| **CD16/32** | - | 93 | Biolegend | 101302 |
| **CD19** | BUV395 | 1D3 | BD Biosciences | 563557 |
| **CD44** | FITC | IM-7 | BD Biosciences | 553133 |
| **CD45** | BUV805 | 30-F11 | BD Biosciences | 568336 |
| **CD62L** | AF700 | MEL-14 | BD Biosciences | 104418 |
| **F4/80** | BUV563 | T45-2342 | BD Biosciences | 749284 |
| **I-A/I-E (MHC-II)** | APC-Cy7 | M5/114.15.2 | Biolegend | 107628 |
| **Ly6C** | BV605 | AL-21 | BD Biosciences | 563011 |
| **Ly6G** | AF700 | 1A8 | Biolegend | 127622 |
| **IFNγ** | FITC | XMG1.2 | BioLegend | 505806 |
| **IL17A** | APC-Cy7 | TC11-18H10.1 | BioLegend | 506940 |
| **Pro-IL1β** | PE-Cy7 | NJTEN3 | Invitrogen | 25-7114-82 |
| **IL10** | PE | JES5-16E3 | Invitrogen | 12-7101-82 |

**Supplementary Table 2.** Anatomic data of female wild-type (WT) control mice and TAC mice treated with IgG or anti-IL12β antibody

| **Parameters** | **Control mice**  **(n=5)** | **TAC+IgG**  **(n=7)** | **TAC+anti-IL12β**  **(n=7)** |
| --- | --- | --- | --- |
| LV Weight (mg) | 65.5±1.61 | 140.91±4.7* | 119.17±6.87^#^**^†^** |
| LA Weight (mg) | 2.74±0.24 | 13.74±2.39* | 4.34±0.66**^†^** |
| Lung Weight (mg) | 111.3±3.67 | 361.44±56.46* | 155.53±16.02**^†^** |
| RV Weight (mg) | 12.46±1.18 | 25.83±1.9* | 18.29±0.95^#^**^†^** |
| RA Weight (mg) | 2.6±0.2 | 4.33±0.35* | 3.27±0.36 |
| Total Heart Weight (mg) | 83.3±2.78 | 184.81±8.64* | 145.07±8.68^#^**^†^** |
| LV weight/BW (mg/g) | 3.72±0.09 | 6.73±0.33* | 5.19±0.2^#^**^†^** |
| LA weight/BW (mg/g) | 0.15±0.01 | 0.66±0.12* | 0.19±0.02**^†^** |
| Lung weight/BW (mg/g) | 6.31±0.17 | 17.42±2.88* | 6.78±0.65**^†^** |
| RV weight/BW (mg/g) | 0.70±0.05 | 1.24±0.11* | 0.8±0.03**^†^** |
| RA weight/BW (mg/g) | 0.15±0.01 | 0.21±0.02 | 0.14±0.01 |

Data are mean ± SEM. *p<0.05 IgG TAC mice compared with the control, #p<0.05 anti-IL12β TAC mice compared with the control, **^†^**p<0.05 compared with IgG TAC mice; BW, body weight.


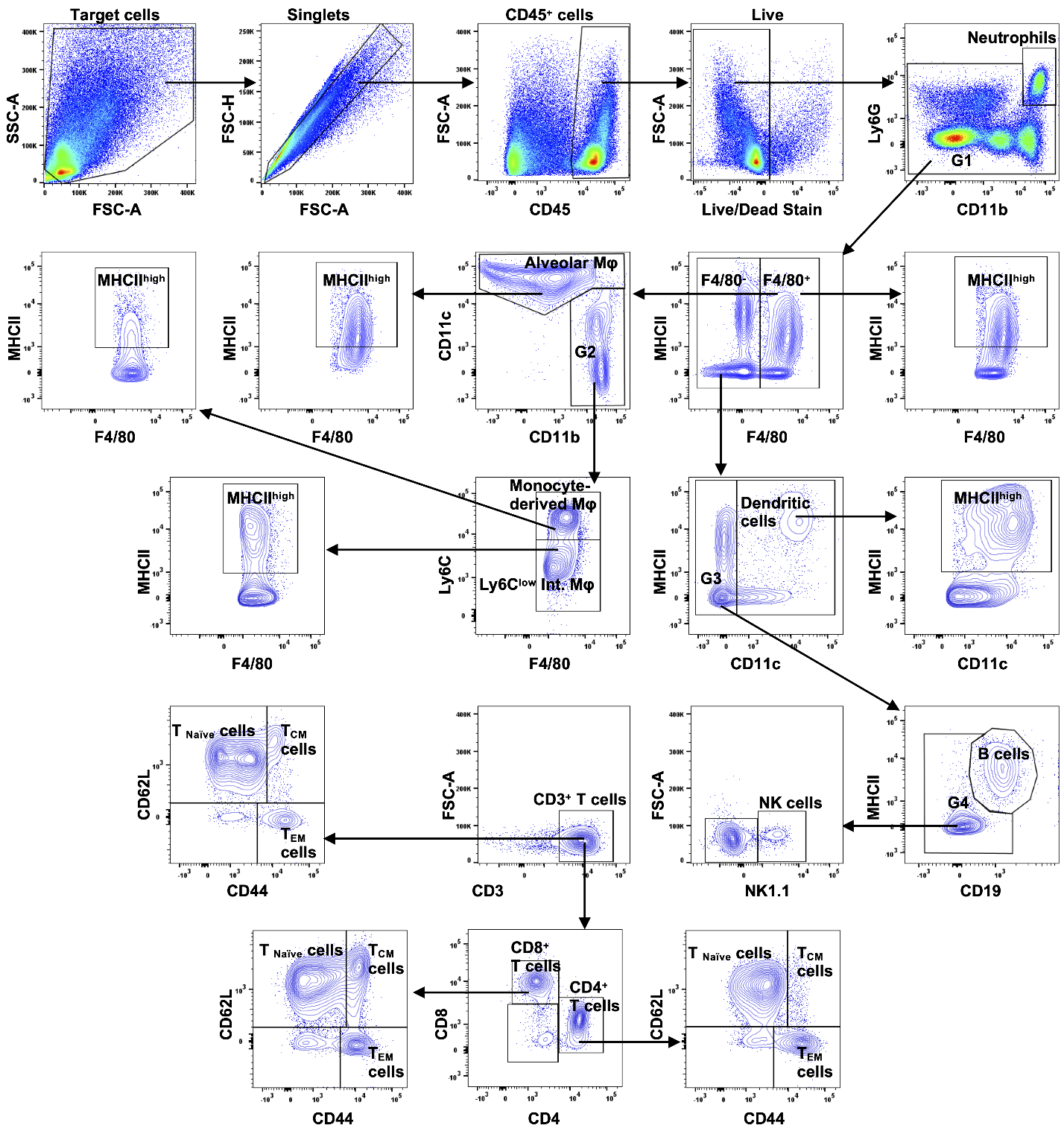


**Supplementary Figure 1:** Flow cytometry gating strategy used for identification of Neutrophils, Macrophages, Dendritic cells, B cells, and T cells in the lung; T_CM_, Central Memory T cells; T_EM_, Effector Memory T cells.


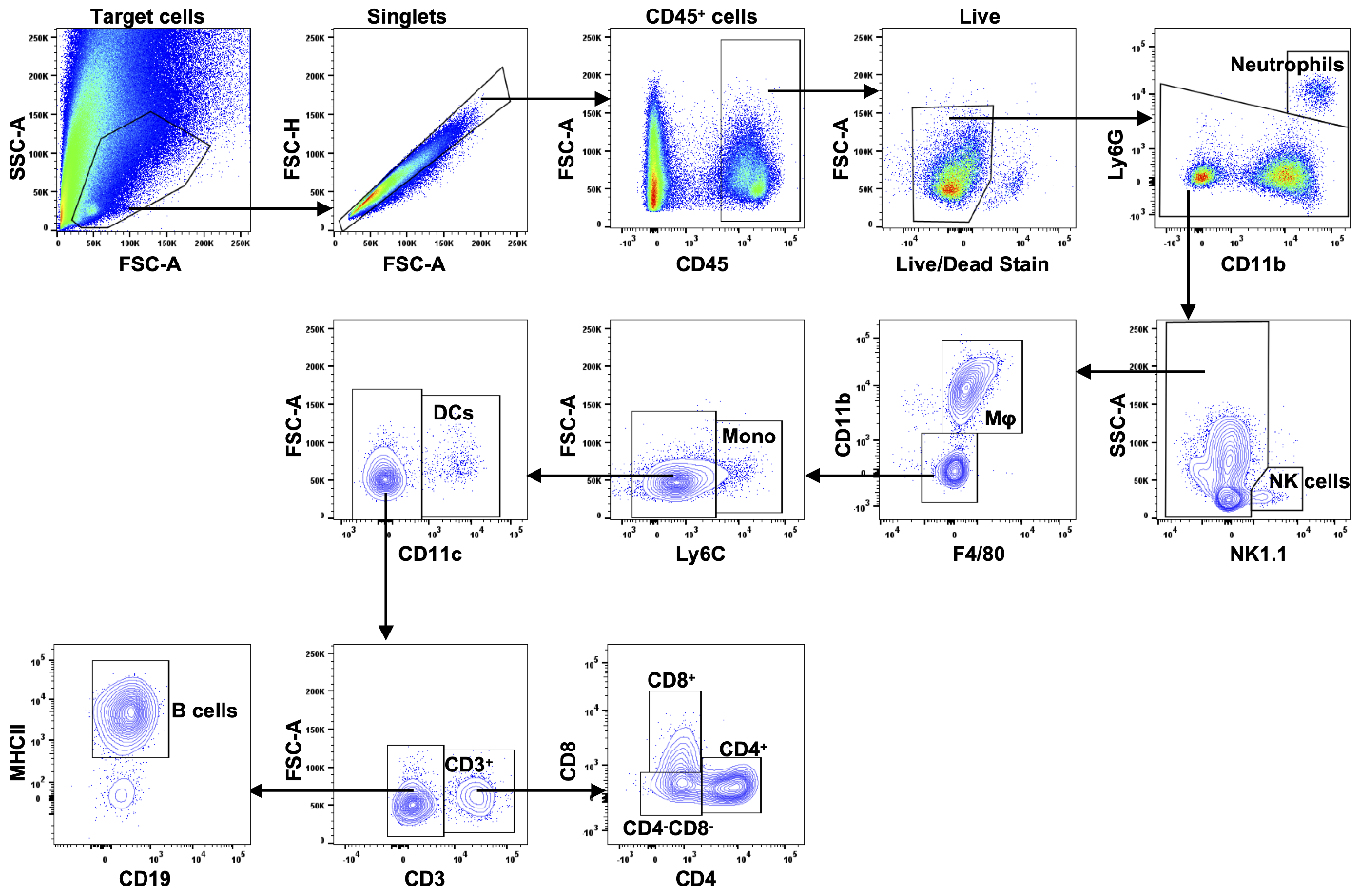


**Supplementary Figure 2:** Flow cytometry gating strategy used for identification of Neutrophils, NK cells, Macrophages, Monocytes, Dendritic cells, T cells, and B cells in the heart; Mφ, Macrophages; Mono, Monocytes; DCs, Dendritic cells.


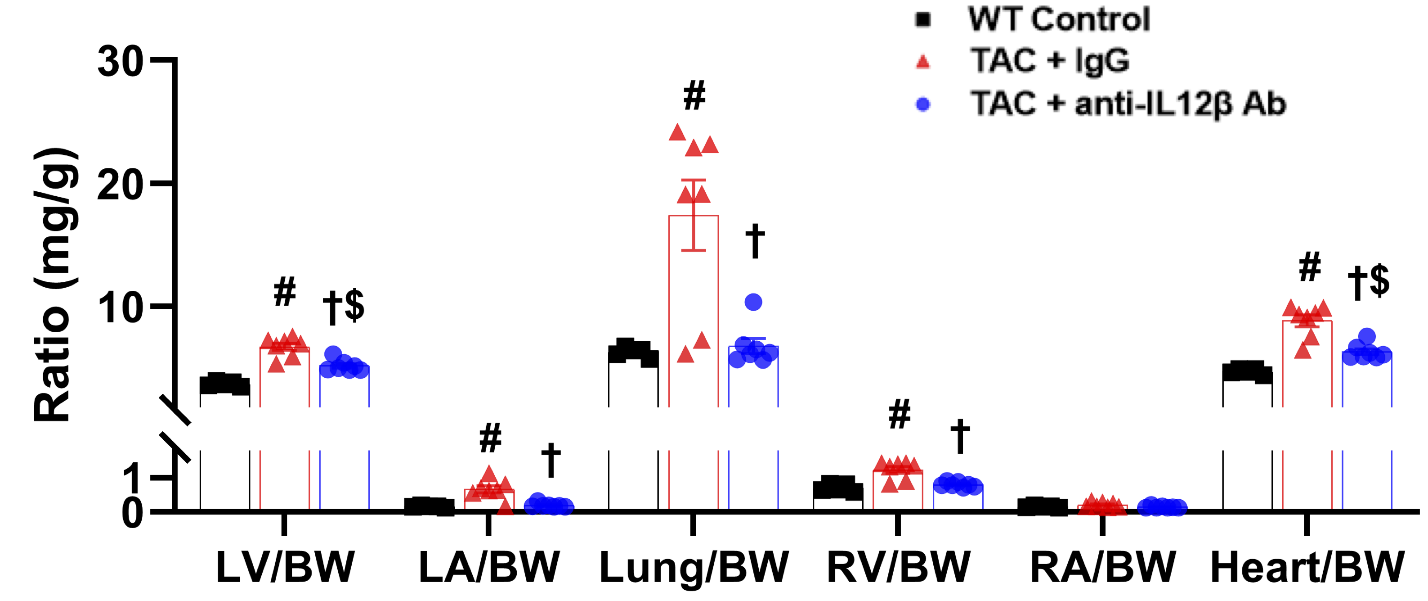


**Supplementary Figure 3:** The ratio of LV, LA, lung, RV, RA, and heart to body weight (BW) of the indicated groups. ^#^p<0.05 IgG-treated TAC mice compared with the control; **^†^**p<0.05 anti-IL12β-treated TAC mice compared with IgG-treated TAC mice; ^$^p<0.05 anti-IL12β-treated TAC mice compared with the control; n=5-7 mice per group.


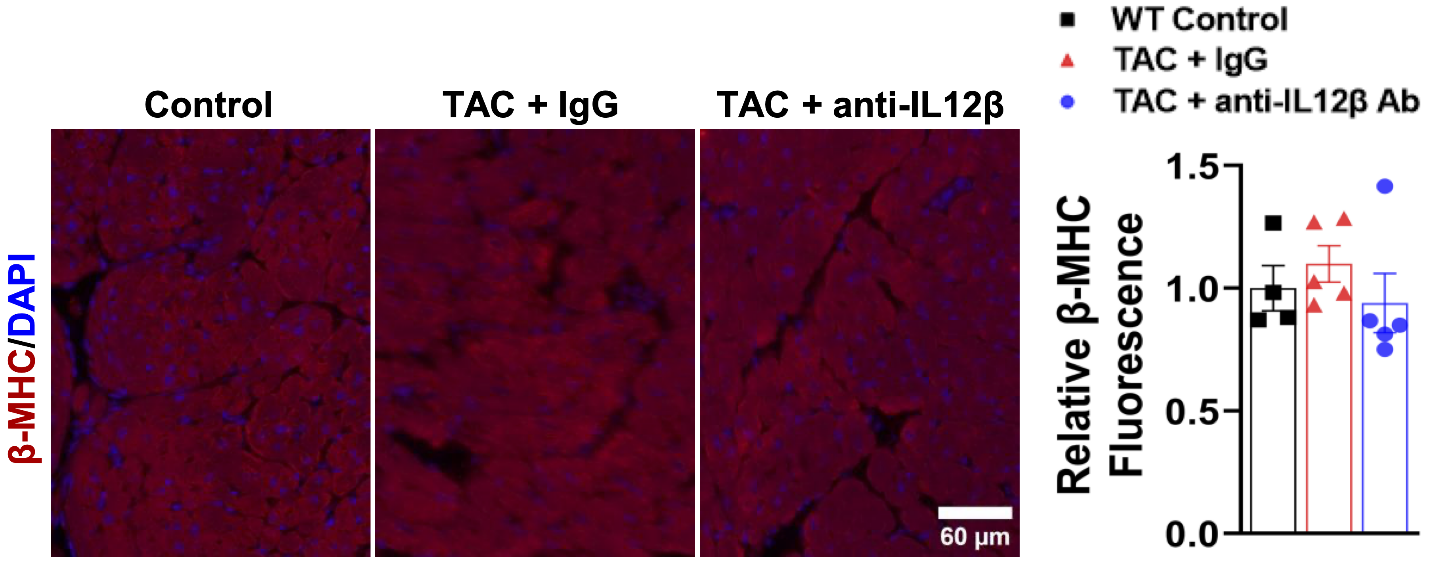


**Supplementary Figure 4:** Representative images and quantification of β-MHC expression in RV tissue. *p<0.05; n=4-5 mice per group.


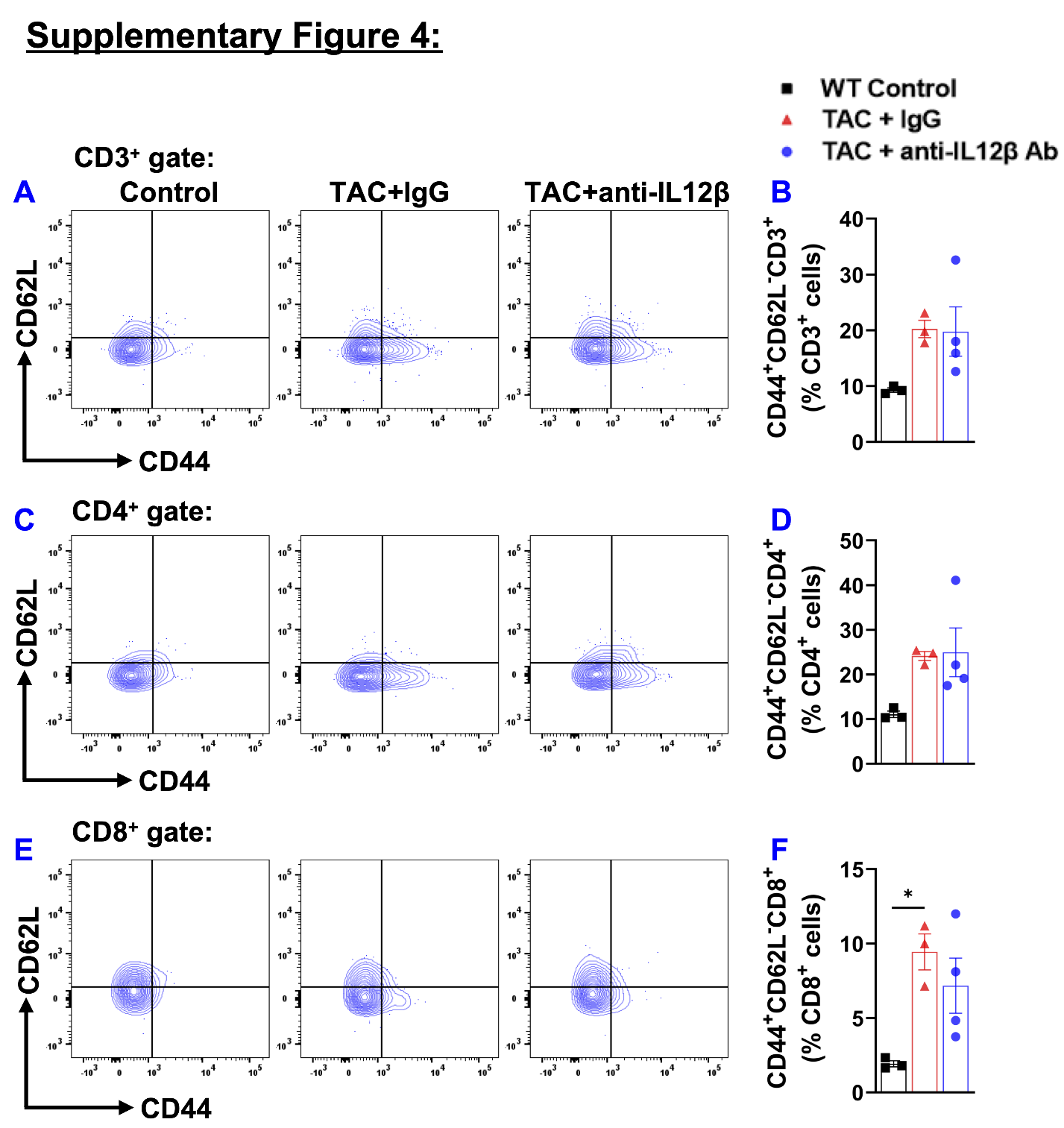


**Supplementary Figure 5:** Flow cytometry plots and quantified data of CD3^+^, CD4^+^, and CD8^+^ T cell activation. *p<0.05; n=3-4 mice per group.


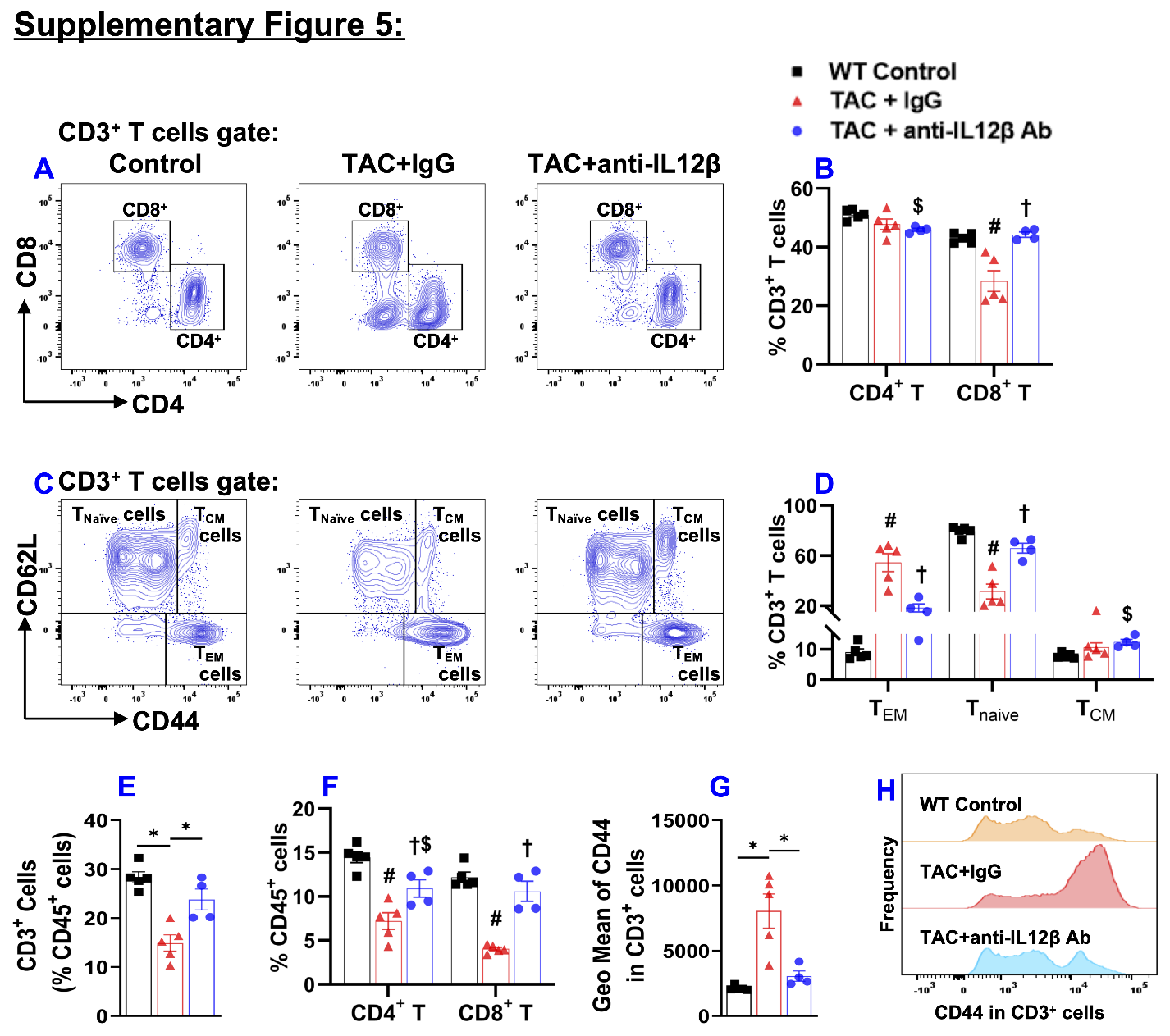


**Supplementary Figure 6:** (A) Flow cytometry plots used for the identification of CD4^+^ and CD8^+^ T cells from CD3^+^ T cells. (B) Quantified data of percentage of CD4^+^ and CD8^+^ T cells within CD3^+^ T cells. (C) Flow cytometry plots for the determination of the activation status of CD3^+^ T cells. (D) Quantified data of the percentage of CD44^+^CD62L^-^ effector memory T cells, CD44^-^CD62L^+^ naïve T cells, and CD44^+^CD62L^+^ central memory T cells within CD3^+^ T cells. (E) Quantified data of percentage of CD3^+^ T cells within CD45^+^ leukocytes. (F) Quantified data of percentage of CD4^+^, and CD8^+^ T cells within CD45^+^ leukocytes. (G) Quantified data of mean fluorescent intensity of CD44 in CD3^+^ T cells. (H) Representative histograms of CD44 expression in CD3^+^ T cells of the indicated groups. *p<0.05; ^#^p<0.05 IgG-treated TAC mice compared with the control; **^†^**p<0.05 anti-IL12β-treated TAC mice compared with IgG-treated TAC mice; ^$^p<0.05 anti-IL12β-treated TAC mice compared with the control; Central Memory T (T_CM_) cells; Effector Memory T (T_EM_) cells; n=4-5 mice per group.
