# Supplementary material for: Pharmacological inhibition of IL12β is effective in treating pressure overload-induced cardiac inflammation and heart failure
Source: Front Immunol. 2025 Aug 15;16:1624940. doi: 10.3389/fimmu.2025.1624940 (PMC12395052; doi:10.3389/fimmu.2025.1624940)
Supplement: Supplementary file 2 [file DataSheet2.pdf]

The International Society for the Advancement of Cytometry (ISAC) has highlighted the importance of including comprehensive methodological information to ensure data reproducibility and reliability. In line with this, Frontiers in Immunology now requires authors to submit a checklist for manuscripts that involve flow or mass cytometry. This checklist helps standardize the reporting process, improving the quality and transparency of published data. By doing so, we support scientific progress, making it easier for other researchers to replicate and validate experiments.

This form should be submitted with any manuscripts using flow or mass cytometry.

Sample/specimen/material description

☐ Total blood

☐ PBMCs

☑ Organ digests

Other____

Did the samples suffer any treatment before or after incubation with the antibodies?

☐ Drug ______________________________________

☑ Cell permeabilization ______________________ Cells were permeabilized with permeabilization buffer (Invitrogen, 00-8333-56) for intracellular cytokines assay.

☐ Dye ______________________________________

☐ Propidium iodine

☐ Not applicable

Other ______________________________________ Cells were stimulated with 1X cell stimulation cocktail (Invitrogen, 00-4970-93) and 1X protein transport inhibitor cocktail (Invitrogen, 00-4980-93) for 2 hours to stimulate cytokines production.

Instrument and antibodies

Name of the Cytometer BD FACSymphonyTM A3 Cell Analyzer (BD Biosciences)

Antibodies and targets	Fluorochrome/ Metal	Catalog number/Company
e.g. anti-CD4	FITC	Cat. XXX/ XXX ltd.
CD3e	BUV737	612771/BD Biosciences

CD4	BUV496	612952
CD8α	BB790	624296
CD11b	BV650	101259
CD11c	BV711	117349
CD16/32	-	101302
CD19	BUV395	563557
CD44	FITC	553133
CD45	BUV805	568336
CD62L	AF700	104418
F4/80	BUV563	749284
I-A/I-E (MHC-II)	APC-Cy7	107628
Ly6C	BV605	563011
Ly6G	AF700	127622
IFNγ	FITC	505806
IL17A	APC-Cy7	506940
Pro-IL1β	PE-Cy7	25-7114-82
IL10	PE	12-7101-82

Data analyses

1. Name of the software FlowJo-v10 software
2. Reference gating strategy in the manuscript or supplementary material

Gating strategy in (eg Figure X) Supplementary Figures 1 and 2
